# Exosomal gene-based predictive model and therapeutic target identification for Alzheimer’s disease: A bioinformatics analysis

**DOI:** 10.1371/journal.pone.0354014

**Published:** 2026-07-20

**Authors:** Lei Ma, Dongfeng Wang, Zhenqiang Li, Gengfan Ye, Maosong Chen

**Affiliations:** Department of Neurosurgery, Ningbo Medical Center Lihuili Hospital, Ningbo University, Ningbo, Zhejiang, China; Al-Azhar University / King Khalid University, EGYPT

## Abstract

**Background:**

Alzheimer’s disease (AD) is a degenerative central nervous system disorder characterized by progressive cognitive and behavioral impairment. As nanoscale intercellular communication vesicles that carry AD-related pathological molecules, exosomes are promising biomarkers and therapeutic carriers for AD. In this study, we downloaded AD-related gene expression profiles and clinical data from the Gene Expression Omnibus (GEO) database (datasets GSE138260, GSE29378, GSE36980, and GSE5281). Through a series of bioinformatics analyses, clinical predictive model construction, pharmacological network analysis, and molecular docking simulations, we developed an exosomal gene-based predictive model for AD pathogenesis and identified potential pharmacological networks and molecular docking targets for AD treatment.

**Materials and methods:**

AD-related gene expression and clinical data were retrieved from the GEO database. Bioinformatics analyses, clinical model construction, drug-gene network analysis, and molecular docking were subsequently performed to explore exosomal gene models for predicting AD pathogenesis, as well as potential pharmacological networks and molecular docking targets for AD therapy.

**Results:**

A five-exosomal-gene predictive model was established, comprising CD44, CXCR4, TUBB, PSMA5, and PSMB3. Pharmacological network analysis of these five genes revealed their significant associations with chelidonine, 2-chloro-1,4-dinitrobenzene, oxazolone, phencyclidine, thioridazine, and etodolac. Further molecular docking simulations identified key binding targets, including R41, Y42, R78, Y79, C77, I88, C97, A98, I96, I72, L70, E67, G103, I91, and T102.

**Conclusions:**

Our comprehensive analyses successfully established a reliable exosomal gene-based model for predicting AD pathogenesis, and identified relevant pharmacological networks and core molecular docking targets, providing novel insights for AD diagnosis and targeted therapy.

## Introduction

Exosomes are small membrane-bound vesicles secreted by eukaryotic cells, with a typical diameter ranging from 40 to 100 nanometers [1]. They are rich in a variety of bioactive molecules, including proteins, lipids, and nucleic acids, which play a key role in cell-to-cell communication and physiological pathological processes. Due to their unique nanoscale structure and biocompatibility, exosomes are becoming a hot spot in biomedical research [2]. Exosomes play an important role in AD research [3]. They are key mediators of cell-to-cell communication and are involved in the pathological process of Alzheimer’s disease. The use of bioinformatics methods, such as sequence analysis and protein structure prediction, can help identify key genes in exosomes and provide important clues for the early diagnosis of Alzheimer’s disease [4]. In this study, we established a model that can predict the gene composition of exosomes in AD through relevant molecular biology analysis and clinical analysis, which is composed of: CD44, CXCR4, TUBB, PSMA5 and PSMB3 [5–7]. The pharmacological network analysis of the above five genes showed that these genes were mainly related to chelidonine, chloro-2, 4-dinitrobenzene, oxazolone, phencyclidine, thioridazine and etodolac. Finally, we further performed molecular docking to obtain possible targets, which are: R41, Y42, R78, Y, 79, C77, I88, C97, A98, I96, I72, L70, E67, G103, I91 and T102.

## Method

### Data downloads

Gene expression profiles and associated clinical annotations of Alzheimer’s disease (AD) were retrieved from the Gene Expression Omnibus (GEO) database. Four datasets (GSE138260, GSE29378, GSE36980, GSE5281) were included in this study. All samples were obtained from human brain tissue.

### Data normalization

Raw expression data were normalized using log_2_ transformation and between-array normalization with the limma package in R. Reproducible code is provided in the supplementary file: data normalization.txt.

### Batch effect correction

Batch effects across the four datasets were eliminated using the ComBat algorithm from the sva package. After correction, integrated expression data were generated for subsequent analysis. Code is available in SVA.txt.

### Differential expression analysis

Differentially expressed genes (DEGs) were identified using the limma package. Thresholds were set as |logFC| > 0.5 and adjusted P value < 0.05. DEGs were visualized using volcano plots and heatmaps. Code is provided in BatchDiff.txt.

### Exosome-related gene collection

Exosome-related genes were downloaded from the GeneCards database (https://www.genecards.org/) with a relevance score ≥ 2 as the filtering criterion [8].

### Principal components analysis (PCA) analysis

Common genes across datasets were identified using PCA and Venn diagram intersection. DEGs were intersected with exosome-related genes to obtain candidate AD‑associated exosomal genes. Code is provided in PCA analysis.txt.

### Functional enrichment analysis

Gene Ontology (GO), Kyoto Encyclopedia of Genes and Genomes (KEGG), and Gene Set Enrichment Analysis (GSEA) were performed to annotate biological functions and pathways.

### Univariate logistic regression analysis

Univariate logistic regression was applied to screen exosomal DEGs significantly associated with AD (P < 0.05). Code is provided in logistic.txt.

### Least Absolute Shrinkage and Selection Operator (LASSO) regression

LASSO regression was performed using the glmnet package with 10‑fold cross‑validation to refine key diagnostic genes. The penalty parameter λ was determined by the minimum cross‑validation error. Code is provided in lasso.txt.

### Support Vector Machine‑Recursive Feature Elimination (SVM‑RFE)

SVM‑RFE was used to identify the optimal gene set with the minimum 10‑fold cross‑validation error for AD classification. Code is provided in Exosome16.SVM.txt and Exosome16.msvmRFEt.txt.

### Random forest analysis

A random forest classifier was constructed with ntree = 500 to calculate gene importance scores (MeanDecreaseGini) and validate the stability of feature selection. Genes with high importance were retained. Code is provided in randomForest.txt.

### ROC curve (Receiver Operating Characteristic) analysis

ROC curve analysis was conducted to evaluate the predictive performance and accuracy of the model. Analyze code: ROC analysis.txt.

### Nomogram

A nomogram was established to estimate the predictive probability of AD and validate model performance. Calibration curves and decision curve analysis (DCA) were also performed. Code is provided in Nomo.txt.

### Immune cell analysis

Immune cell infiltration scores were calculated to evaluate the correlation between the gene signature and the immune microenvironment in AD.

### Network pharmacological analysis

The relationship between genes and candidate drugs was analyzed using the DSigDB database (https://dsigdb.tanlab.org/DSigDBv1.0/) [9].

### Molecular docking

Molecular docking was performed using the CB-Dock2 platform (https://cadd.labshare.cn/cb-dock2/index.php) to predict binding sites and affinity between candidate compounds and target proteins [10–12].

### Ethics approval and consent to participate

This study is an open-source data analysis and does not require ethical review.

## Result

**Fig 1:**
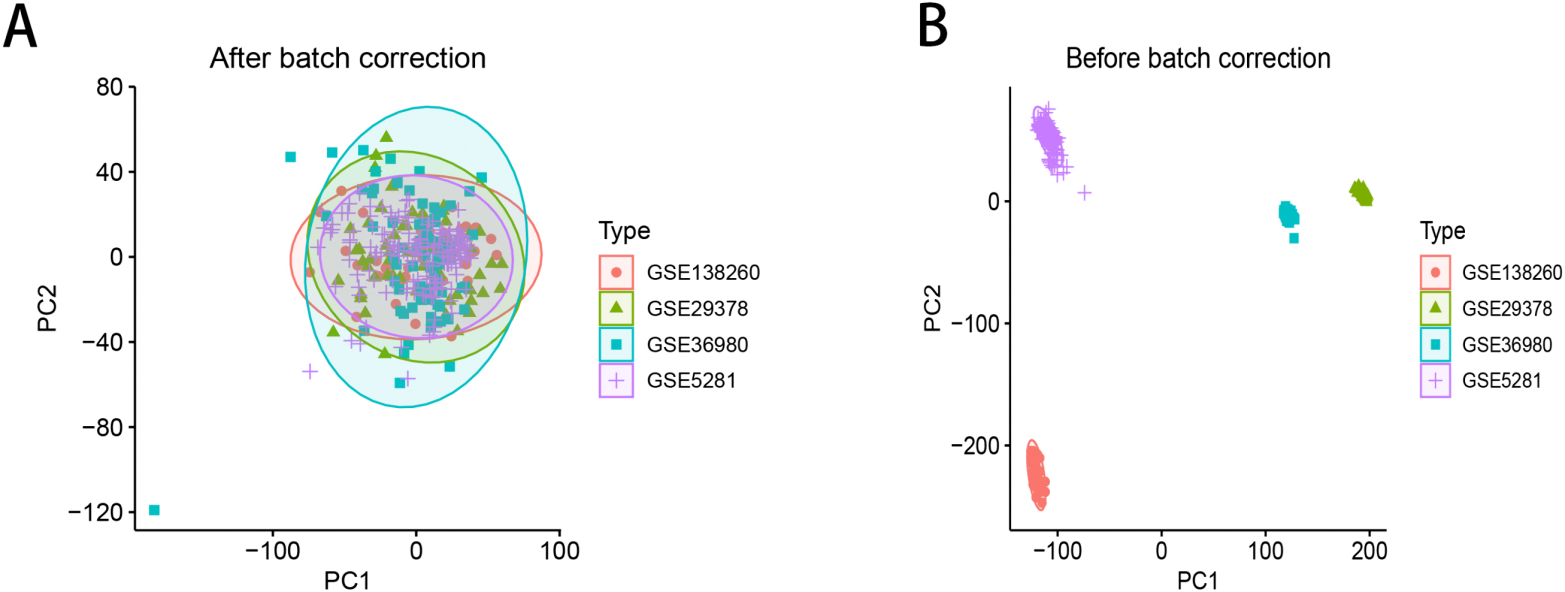
PCA analysis was performed on four AD datasets. A. PCA analysis, after batch correction; B. PCA analysis after batch correction. Different colors represent different datasets, and the distribution of the datasets can be judged based on the color patterns.

Download Alzheimer’s related data, including gene expression data and clinical data, including (GSE138260, GSE29378, GSE36980, and GSE5281) from the GEO database. PCA analysis of its common genes (Fig 1). Exosome-related genes were collected through relevant literature and databases (https://www.genecards.org/).

**Fig 2:**
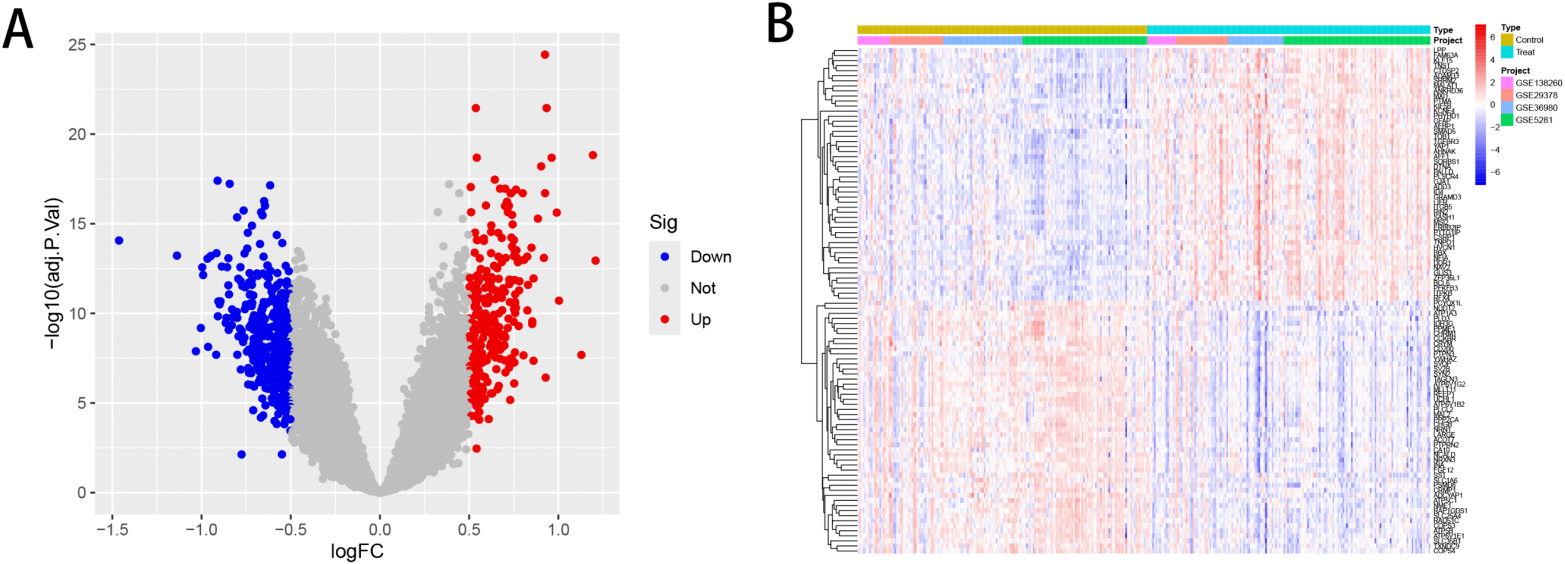
Differential gene screening and visual analysis. A. Volcano plot. Red represents overexpressed genes and blue represents underexpressed genes. B. Heat map. Red represents overexpressed genes and blue represents underexpressed genes.

**Fig 3:**
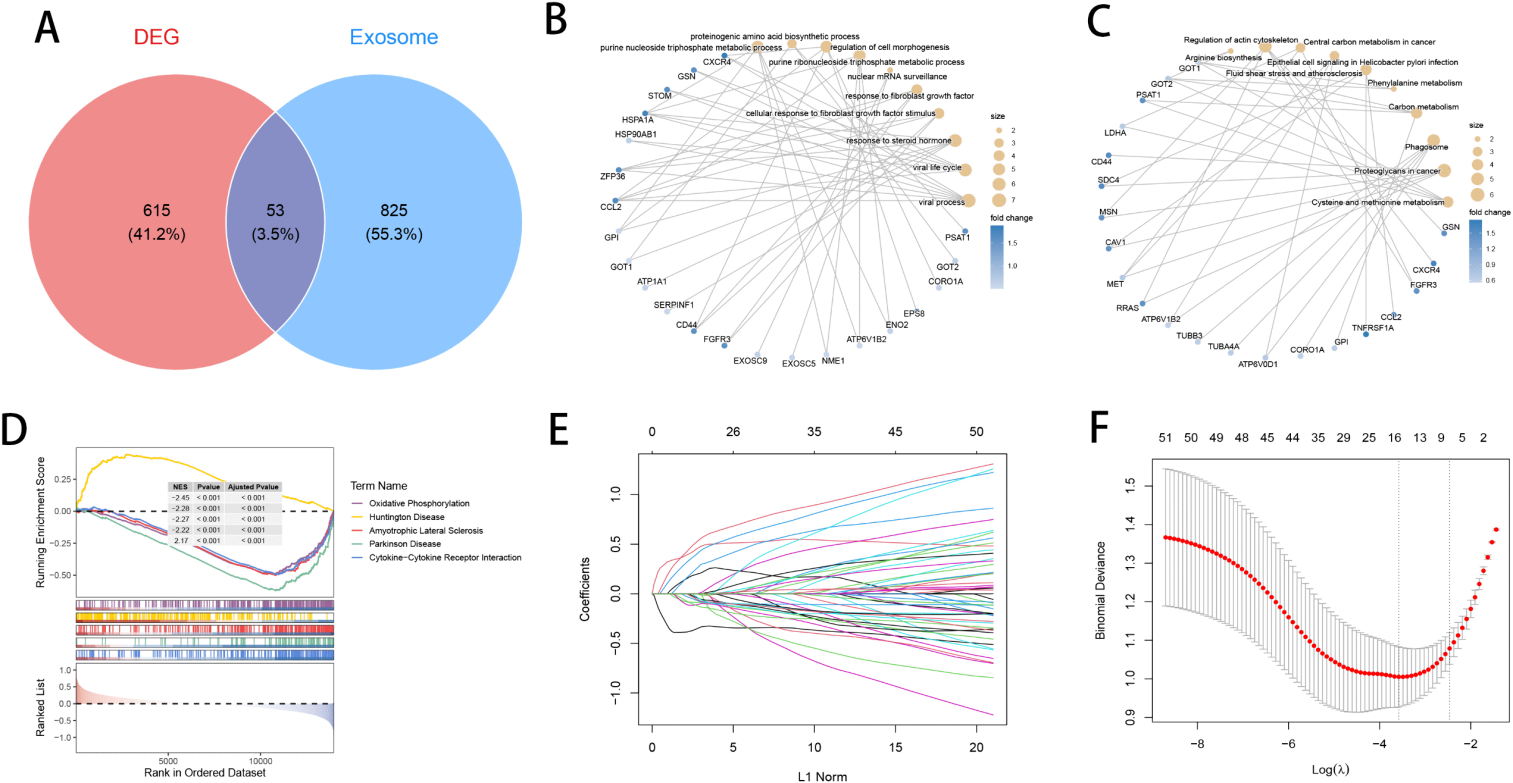
Exosomes Gene Screening and Functional Analysis. A. Venn diagram, analysis of conjugated genes, A total of 53 co-expressed genes were identified; B. Biological function analysis. Yellow circles indicate gene size; larger circles represent a larger size (Blue boxes represent fold change; a darker color indicates a larger value); C. KEGG pathway analysis; D. GSEA enrichment analysis; E. Logistic analysis; F. LASSO analysis.

**Fig 4:**
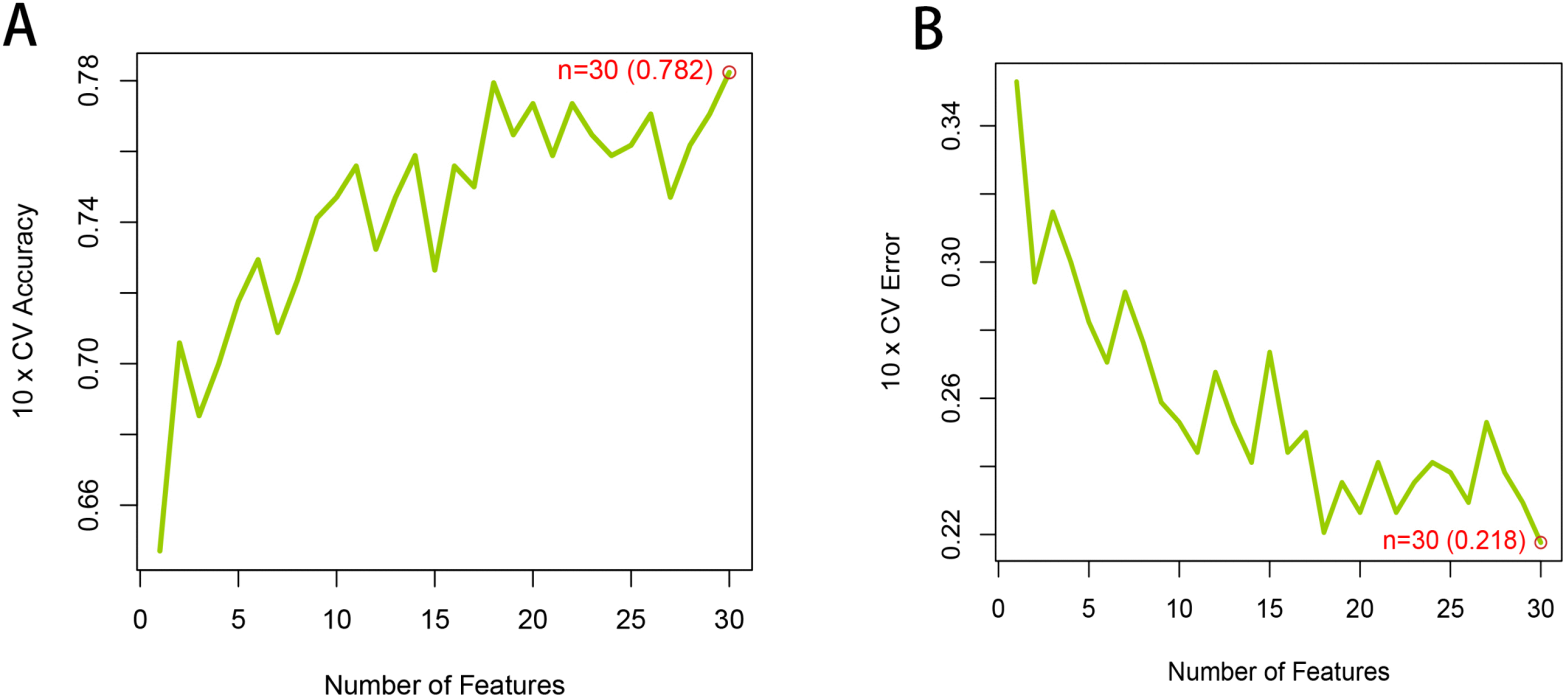
SVM machine learning screens disease signatures. The abscissa represents the number of features, and the ordinate represents 10× CV Accuracy.

**Fig 5:**
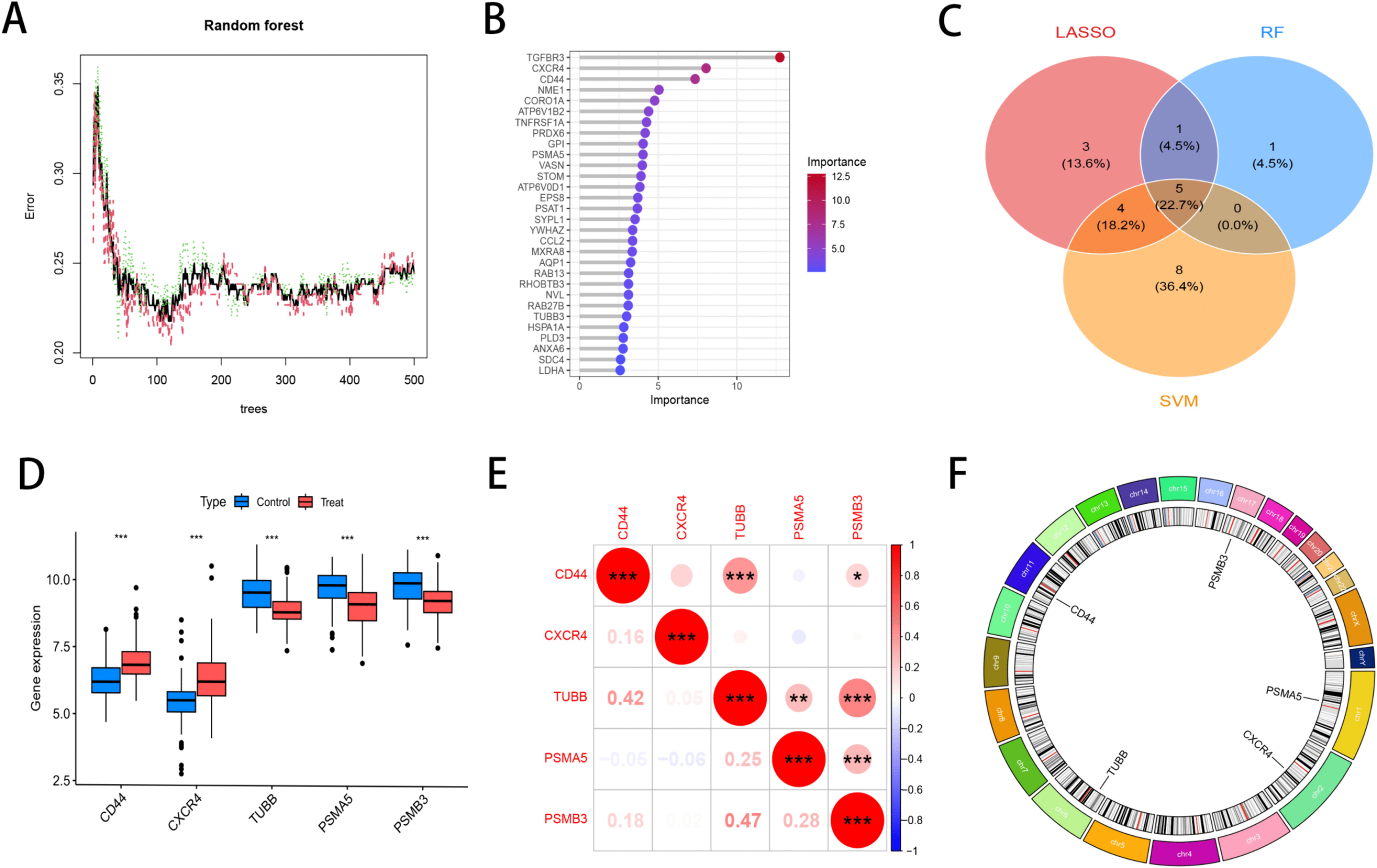
Random forest tree screening of disease characteristic genes. A is random forest; B. Important index of each gene; C. Venn diagram; D gene expression analysis, E. Co-expression heat map correlation analysis; F. Gene expression circle diagram.

**Fig 6:**
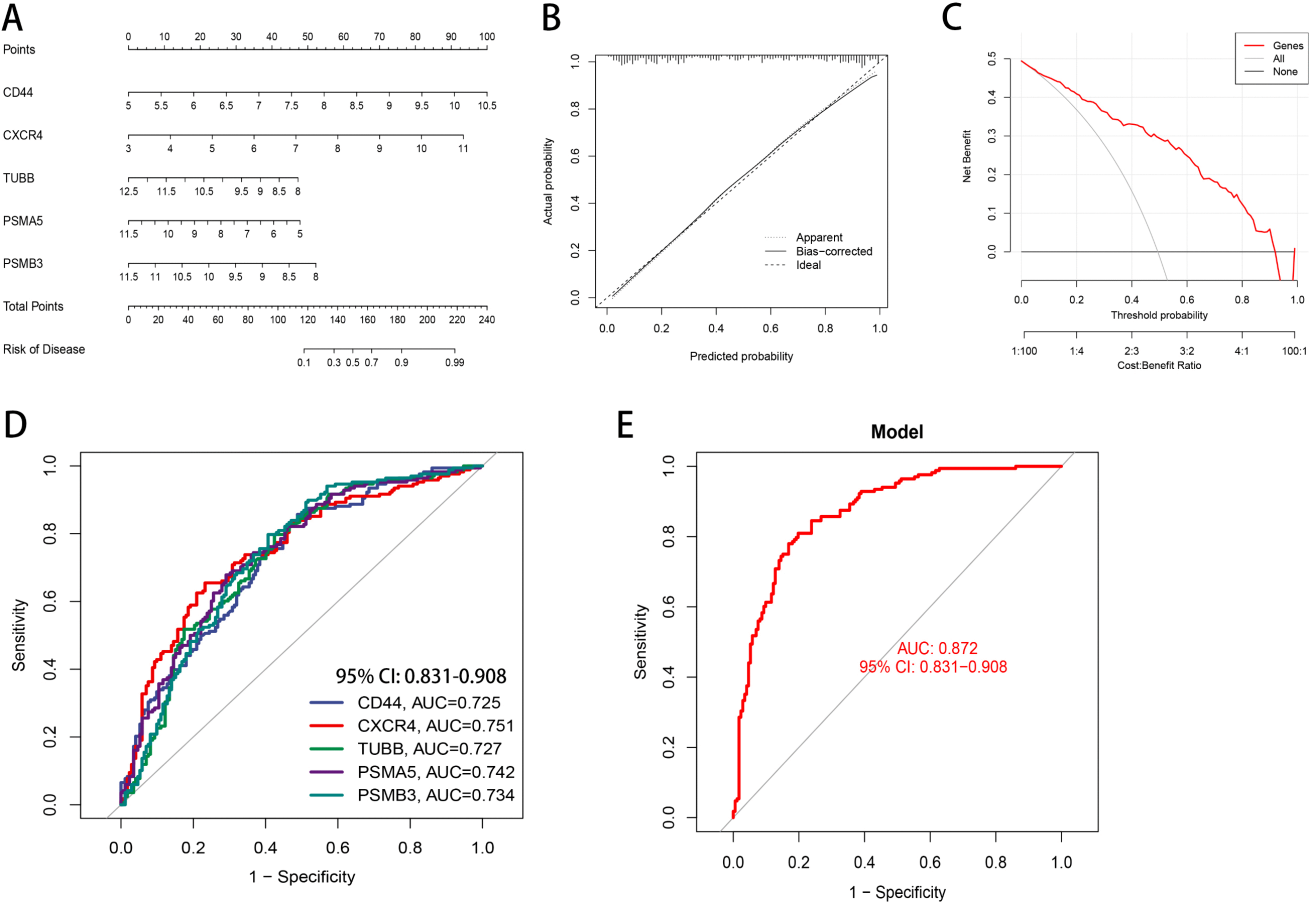
Clinical Correlation Analysis. A. Nomogram; B. Calibration diagram; C. DCA diagram; D. Independent validation ROC curve; E. Cross-validation ROC curve.

**Fig 7:**
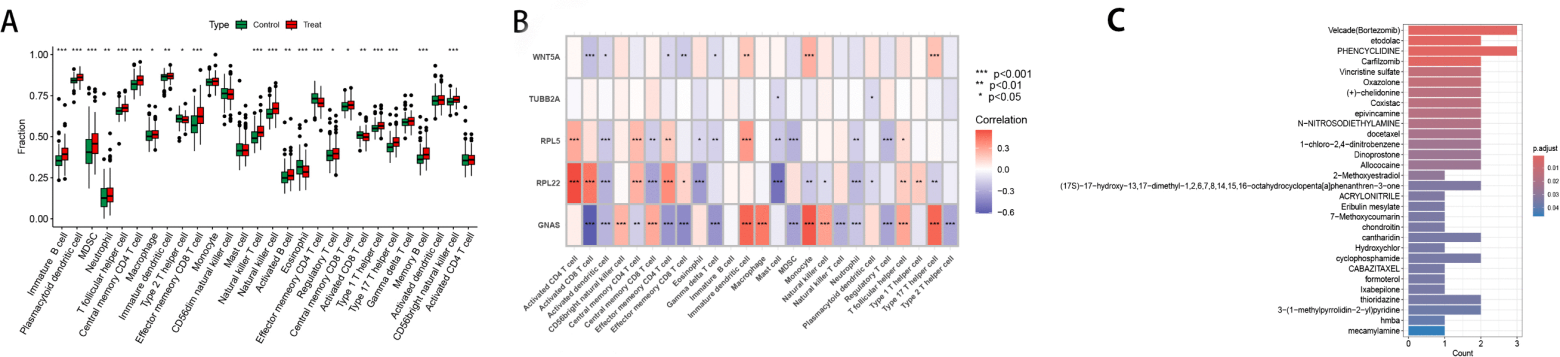
Immune cells correlation analysis. A. the composition of immune cells in the sample; B. predict the relationship between model-related genes and immune cells; C. Predict the individual immunological-relevant functions of the model.

**Fig 8:**
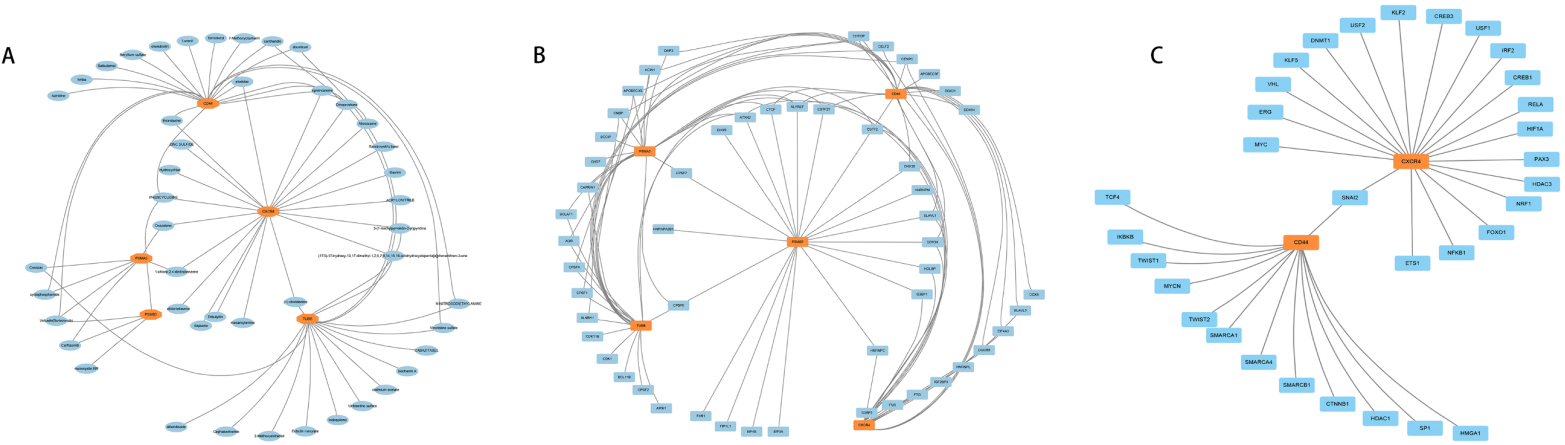
Hub genes regulatory network. A. Drug Regulatory Network; B. RBP regulatory network (RNA-binding proteins); C.TF transcription factor regulatory network.

**Fig 9:**
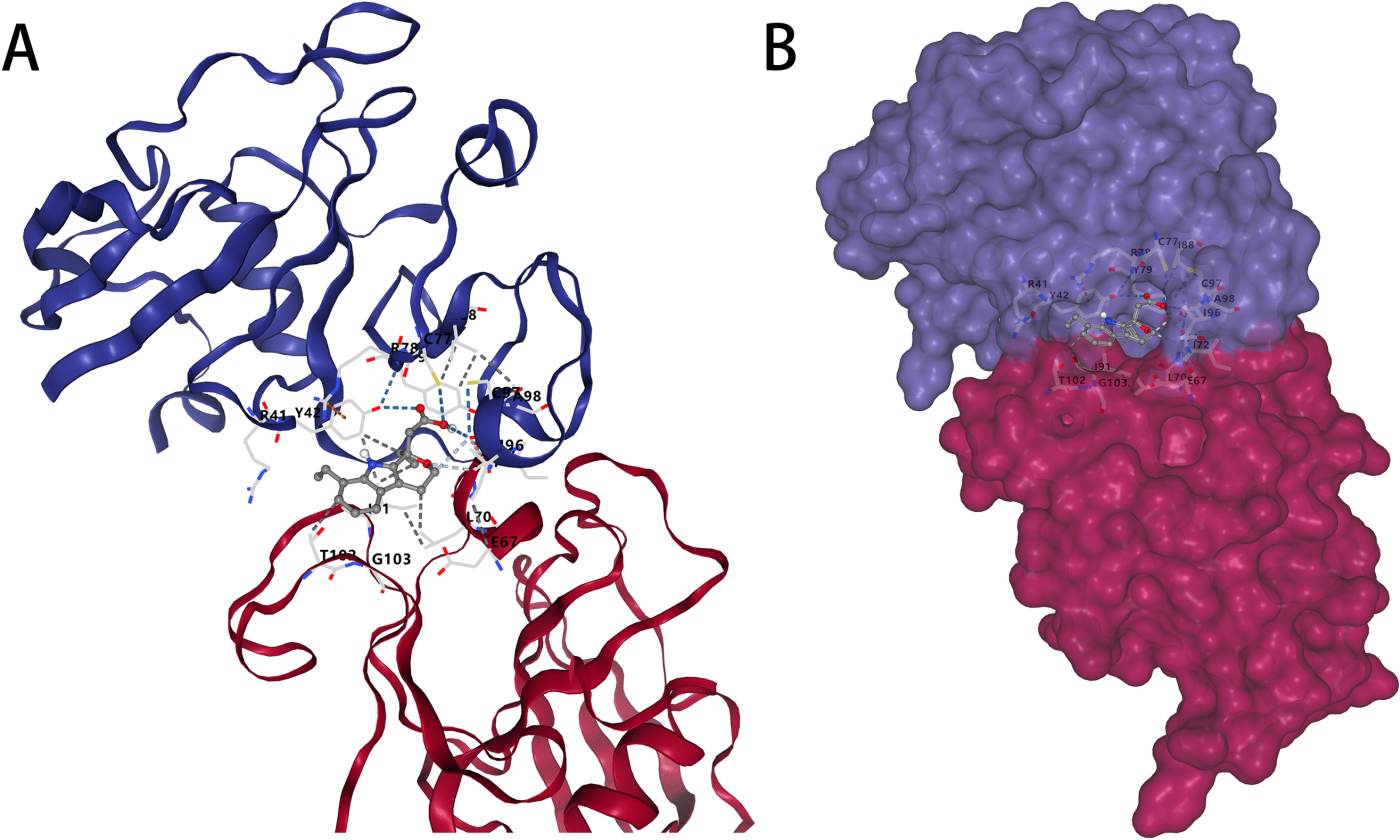
Molecular docking simulation. A. Simple structure. B. Three-dimensional structure. Docking targets: R41, Y42, R78, Y, 79, C77, I88, C97, A98, I96, I72, L70, E67, G103, I91 and T102.

Through differential analysis, we identified 668 differentially expressed genes and visualized them using a volcano plot and a heatmap (Fig 2). Venn diagram analysis identified the four AD samples in this study had 53 exosome-related conjugated genes. Biological function analysis showed that the genes in this study were mainly enriched in response to steroid hormone, cysteine and methionine metabolism, Oxidative phosphorylation, Huntington disease, and Amyotrophic lateral sclerosis (Fig 3A–3D). Logistic analysis was used to explore the correlation between these exosome genes and AD, and the results obtained were further analyzed by Lasso regression analysis and support vector machine machine learning (SVM). Lasso regression analysis yielded 16 strongly correlated genes, namely NME1, TGFBR3, TNFRSF1A, PRDX6, CD44, PLD3, CXCR4, PSMA5, VASN, SERPINF1, LDHA, NEU1, WDR46, CORO1A, NVL and MET. There were 30 related genes obtained by SVM machine learning, which were: CXCR4, CD44, VASN, TUBA4A, AQP1, RAB13, MSN, ATP6V1B2, PSMA5, TGFBR3, CAV1, HSPA1A, VIM, TUBB3, GOT2, MET, NVL, NME1, PSAT1, FOXO1, LDHA, CLIC1, TNFRSF1A, PRDX6, PLD3, RHOBTB3, WDR46, GPI, EPS8 and GSN (Figs 3E, 3F and 4). Therefore, we also verified the disease characteristics of random forest tree screening with the results of logistic analysis, and obtained the following strongly correlated genes (Fig 5A and 5B). By Venn analysis, the conjugated genes were screened out from SVM, LASSO regression and RF, finally five genes were obtained: CD44, CXCR4, TUBB, PSMA5 and PSMB3 (Fig 5C, 5D, and 5F). Clinical analysis showed that CD44, CXCR4, TUBB, PSMA5 and PSMB3 could constitute clinical analysis models. Both nomogram and ROC analysis (Independent validation and Cross-validation) showed some accuracy of the clinical model (Fig 6). Immunoassay showed that the clinical models analyzed in this analysis were mainly strongly correlated with immune cells such as B cell, MDSC, CD4, and CD8. These genes were notably associated with Velcade and etodolac (Fig 7). We carried out the drug regulatory network, RBP regulatory network and TF transcription factor regulatory network of the core genes of the model to obtain the relevant drug regulatory network and related functional genes of these genes (Fig 8). The pharmacological network analysis of the above five genes showed that these genes were mainly related to chelidonine, chloro-2, 4-dinitrobenzene, oxazolone, phencyclidine, thioridazine and etodolac (Fig 8A). Finally, we predicted the molecular docking of the model-related genes. Docking targets: R41, Y42, R78, Y, 79, C77, I88, C97, A98, I96, I72, L70, E67, G103, I91 and T102. These docking residues were located within the functional domains of the target proteins and contributed to stable ligand binding (Fig 9). Detailed information on key amino acid residues for molecular docking is shown in S1 Table.

## Discussion

Exosomes have made significant progress in the study of AD. As key mediators of cell-to-cell communication, exosomes have multiple properties, such as anti-apoptosis, anti-inflammatory, etc., which help inhibit the neuroinflammatory cascade in the pathogenesis of AD, promote Aβ clearance, and reduce Aβ content in the brain, thereby improving cognitive function. In addition, exosomes are abundant and easy to quantify, store, and transport, providing a new perspective for the treatment of AD. In recent years, more and more research teams have begun to pay attention to the application of exosomes in the treatment of AD, and have achieved a series of important results, bringing new hope to AD patients. In this study, we are committed to constructing an exosomal gene model that can predict AD relatively accurately, which is composed of: CD44, CXCR4, TUBB, PSMA5 and PSMB3 [3,4,13–15]. We select datasets from the GEO database that can be used for research, and we need not only gene expression data but also corresponding clinical data. Finally, we screened four datasets: GSE138260, GSE29378, GSE36980, and GSE5281. In this study, three machine learning algorithms, namely LASSO regression, SVM, and random forest, were combined to screen core exosomal signature genes of AD in a complementary manner. LASSO was used for sparse preliminary screening of high-dimensional gene data; SVM was applied to mine nonlinear associations in high-dimensional and small-sample scenarios; random forest was adopted to validate, rank candidate genes, and quantify their importance. The intersection of the genes identified by the three algorithms was taken to construct a multi-level screening system named “preliminary screening-fitting-validation” [16–18]. Finally, reliable and specific core genes including CD44, CXCR4, TUBB, PSMA5, and PSMB3 were identified. This strategy has been supported by relevant published studies. Through differentiation analysis and correlation analysis, we obtained 5 genes that are not only differentially expressed but also have certain clinical relevance, which are composed of clinical models that can well predict the onset of AD. The ROC curve values of the clinical models were all above 0.70, indicating that the prediction accuracy of the models was moderate and stable diagnostic performance. CD44, a receptor for hyaluronic acid, is upregulated in patients with AD and exerts protective effects against AD by regulating V-ATPase to ameliorate endolysosomal dysfunction and reduce protein toxicity and neurotoxicity [19]. In AD, the expression of CD44 and its splice variants (CD44V3, CD44V6, CD44V10) is significantly higher in the hippocampus of patients than in non-AD controls. CD44S is mainly localized to neuritic plaques and astrocytes, while CD44V3, CD44V6, and CD44V10 are predominantly concentrated in neurons. Aβ peptides can induce the expression of CD44V6 and CD44V10, and CD44V10 is causally associated with Aβ-induced neuronal toxicity and cell death. Inhibition of CD44V10 protects neurons, representing a promising novel neuroprotective therapeutic strategy for AD [20].

CXCR4, a highly conserved seven-transmembrane G protein-coupled receptor, forms a signaling axis with its specific ligand CXCL12 that plays a critical regulatory role in the pathological progression of AD. This pathway is involved not only in neurotransmission, synaptic plasticity, and neuroinflammation in the central nervous system but also mediates the regulation of multiple AD-related downstream signaling pathways including MAPK, PI3K-AKT, and NF-κB via Gi proteins. Studies have shown that the expression of CXCL12/CXCR4 is significantly downregulated in the brains of AD patients and animal models, disrupting normal neuron-glia communication and leading to synaptic dysfunction and impaired learning and memory. It is also closely associated with pathological alterations such as microglial activation, neuroinflammation, and excitotoxicity, mediating core processes including β-amyloid deposition, Tau hyperphosphorylation, and cognitive decline. These findings suggest that the CXCL12/CXCR4 axis may serve as a potentially important target for early prediction, mechanistic research, and targeted therapy of AD [21].

TUBB (β-tubulin), a key component of the cytoskeleton, has been identified as a core biomarker in urinary diagnostic models for the mild cognitive impairment (MCI) stage of AD and is also a critical hub gene linked to AD. Its protein structure and post-translational modifications are significantly altered in the brain tissue of AD patients. By affecting microtubule assembly and stability, neuronal structural integrity, and axonal transport, TUBB contributes to neuronal damage and cognitive decline during AD pathogenesis. Characteristic changes in urinary TUBB are detectable as early as the MCI stage, indicating its potential noninvasive diagnostic value for the early identification of preclinical AD pathological changes [22].

PSMA5 is a key circadian rhythm-related biomarker in AD. Its expression is decreased in Aβ-induced neuronal injury, and it participates in AD pathogenesis by modulating the ubiquitin-proteasome system, immune microenvironment, and circadian homeostasis [23].

In a Mendelian randomization study integrating brain proteomic and genetic data, PSMB3 showed no causal association with AD. Instead, it acts as a potential pathogenic target for amyotrophic lateral sclerosis and is involved in neuronal injury and degeneration by regulating proteasome function [24].

Immunoassay also showed that the model group constructed this time was closely related to immune cells such as B cells and T cells. Further exploration of the RNA binding proteins and transcriptional regulatory networks of the model genes revealed that CD44 and CXCR4 were membrane receptors of this model and their related gene networks were obtained. The five genes that make up the model were analyzed by pharmacological network. These genes were mainly enriched in pathways related to neuroinflammation and proteasome function. Finally, we further performed molecular docking to obtain possible targets, which are: R41, Y42, R78, Y, 79, C77, I88, C97, A98, I96, I72, L70, E67, G103, I91 and T102. Through bioinformatics analysis, this study obtained a model that can predict AD, and further conducted pharmacological network analysis and molecular docking, which is expected to obtain more ways to treat AD. Despite its moderate and stable predictive value, the 5-gene exosome model still faces clinical feasibility, potential risks, and translational barriers. First, the lack of standardized exosome detection methods limits large-scale clinical application. Second, over-reliance on gene expression data may cause non-specific signals, misdiagnosis, or psychological burden. Third, the absence of experimental and external validation, high costs, and unclear in vivo functions impede clinical translation. Further validation and standardization are needed before clinical use. Although there are many AD samples selected in this study, they are not from the same institution after all. If there are more and better datasets in the future, the accuracy of bioinformatics research should be further increased. In addition, due to the tight research funding, we did not conduct relevant experimental verification. A notable additional limitation is the lack of external validation of the predictive model in independent cohorts, which is constrained by the limited accessibility to well-annotated AD brain tissue gene expression datasets—brain tissue specimens from AD patients are rare and highly restricted in access due to ethical sampling requirements and practical collection challenges. To mitigate this limitation, we performed multiple rigorous internal validation approaches (stratified 10-fold cross-validation, independent train-test splitting, and 1000-time bootstrapping) to verify the model’s stability and avoid overfitting. The above are all deficiencies of this study, and we plan to address these limitations in subsequent studies by collaborating with multi-center clinical institutions to collect independent AD brain tissue samples for external validation and conducting in vitro and in vivo experimental verification of the core gene signature and promising pharmacological candidates (e.g., etodolac).

## Conclusion

Our study demonstrates that exosomal genetic signatures hold significant potential for Alzheimer’s disease (AD) risk prediction and therapeutic development. By integrating multi-omics data from four independent cohorts, we established a stable moderate accuracy five-gene predictive model (CD44, CXCR4, TUBB, PSMA5, and PSMB3) with validated diagnostic accuracy (AUC » 0.70). Notably, pharmacological network analysis revealed novel interactions between these exosomal biomarkers and repurposable drug candidates, including etodolac and thioridazine, while molecular docking identified critical binding residues (R41, Y42, C77, etc.) that may guide targeted drug design.

These findings collectively suggest three key advances: 1) Exosomal genes serve as dual-functional biomarkers for both AD diagnosis and treatment monitoring; 2) The identified drug-gene network provides a framework for combinational therapy development; 3) Computational prediction of ligand-receptor interactions enables computationally predicted binding sites for future targeted drug research of AD-related pathways. This integrated approach addresses current limitations in AD biomarker discovery by bridging genomic profiling with translational pharmacology.

The successful identification of immune-associated correlations (particularly with B cells and CD4+/CD8+ T cells) further underscores the interplay between exosome-mediated neuroinflammation and AD progression. Moreover, the construction of TF-RBP regulatory networks elucidates the epigenetic mechanisms underlying exosomal gene dysregulation in AD pathogenesis.

However, several limitations warrant consideration. The retrospective nature of GEO datasets introduces potential cohort heterogeneity bias, and experimental validation of the predicted drug-target interactions remains essential. Future studies should: 1) Validate the model in prospective clinical cohorts with cerebrospinal fluid-derived exosomes; 2) Test prioritized compounds (e.g., chelidonine, oxazolone) in AD animal models; 3) Explore exosomal miRNA/protein cargoes complementary to the genomic signature. Additionally, functional characterization of the docking targets (e.g., Y42 phosphorylation sites) could refine therapeutic specificity.

We searched the relevant literature to investigate the mechanistic relationship between the aforementioned drugs and AD.

Oxazolone, with its lead candidate 15E highly specific to Aβ oligomer/fibril formation, allows early Alzheimer’s disease diagnosis. It can precisely release valproic acid in the disease microenvironment, inhibiting and degrading Aβ fibrils, destroying fibril structure, and reducing Aβ-induced neurotoxicity. This oxazolone-based photosensitive cage combines diagnosis and therapy [25].

In Alzheimer’s patients, NMDA receptor phencyclidine-binding sites (labeled by [^3^H]MK-801) are most severely impaired, with a 34% average reduction in binding capacity in the hippocampal CA1 region. This reduction likely reflects lower receptor density and is unrelated to neuronal loss, plaques, or tangles [26,27].

Thioridazine, a typical antipsychotic, relieves psychosis in Alzheimer’s patients by modulating serotonin and dopamine pathways, indirectly reducing associated skin-picking self-injury. While complete symptom remission was reported in one case, it lacks clinical trial support, raises cerebrovascular risk and adverse effects, and does not directly target the core pathology of Alzheimer’s disease [28].

This study establishes a novel analytical framework in AD management—from conventional single-target approaches to exosome-centric systems medicine. Our computational framework not only accelerates biomarker discovery but also provides actionable insights for developing multi-target therapies against this complex neurodegenerative disorder.

## Supporting information

S1 Table Detailed information of key amino acid residues for molecular docking. Detailed molecular docking amino acid residue information for core target proteins.
(DOCX)

S1 File data normalization.txt. R script for gene expression data normalization.
(TXT)

S2 File BatchDiff.txt. Code for dataset batch effect correction.
(TXT)

S3 File Differential expression analysis of data.txt. Script for differentially expressed gene screening and visualization.
(TXT)

S4 File diff.txt Auxiliary code for differential gene analysis.
(TXT)

S5 File diffGeneExp.txt. Script for extracting DEG expression matrix.
(TXT)

S6 File Exosome genes.csv. List of screened exosome-related genes.
(CSV)

S7 File PCA analysis.txt. Code for PCA and Venn intersection analysis.
(TXT)

S8 File logistic.txt. Script for univariate logistic regression screening.
(TXT)

S9 File lasso.txt. LASSO regression analysis code with cross-validation.
(TXT)

S10 File Exosome16.SVM.txt. Primary SVM-RFE feature screening script.
(TXT)

S11 File Exosome16.msvmRFEt.txt. Auxiliary SVM-RFE analysis script.
(TXT)

S12 File randomForest.txt. Random forest gene importance evaluation code.
(TXT)

S13 File ROC analysis.txt. Script for ROC curve model performance evaluation.
(TXT)

S14 File Nomo.txt. Code for nomogram, calibration and DCA analysis.
(TXT)

S15 File all.txt. Integrated master script for full bioinformatics workflow.
(TXT)

## Abbreviations

AD: Alzheimer’s disease
GSEA: gene set enrichment analysis
ROC: receiver operating characteristic
KEGG: Kyoto Encyclopedia of Genes and Genomes
GO: Gene Ontology Analysis
