# Supplementary material for: Exosomal gene-based predictive model and therapeutic target identification for Alzheimer’s disease: A bioinformatics analysis
Source: PLoS One. 2026 Jul 20;21(7):e0354014. doi: 10.1371/journal.pone.0354014 (PMC13384285; doi:10.1371/journal.pone.0354014)
Supplement: S1 Table — Detailed molecular docking amino acid residue information for core target proteins. (DOCX) [file pone.0354014.s001.docx]

Table S1 Detailed information of key amino acid residues for molecular docking

| Amino acid residue | Corresponding core protein | PDB ID | Functional domain | Conservation score |
| --- | --- | --- | --- | --- |
| R41 | CD44 | <https://doi.org/10.2210/pdb1POZ/pdb> | Extracellular domain | 0.92 |
| Y42 | CD44 | <https://doi.org/10.2210/pdb1POZ/pdb> | Extracellular domain | 0.9 |
| C77 | CXCR4 | <https://doi.org/10.2210/pdb5LFF/pdb> | Extracellular domain | 0.88 |
| L70 | CXCR4 | <https://doi.org/10.2210/pdb5LFF/pdb> | Extracellular domain | 0.85 |
| I72 | CXCR4 | <https://doi.org/10.2210/pdb5LFF/pdb> | Extracellular domain | 0.87 |
| R78 | TUBB | <https://doi.org/10.2210/pdb3QNZ/pdb> | Core domain | 0.95 |
| Y79 | TUBB | <https://doi.org/10.2210/pdb3QNZ/pdb> | Core domain | 0.94 |
| E67 | PSMA5 | <https://doi.org/10.2210/pdb1FUT/pdb> | Catalytic domain | 0.91 |
| I88 | PSMA5 | <https://doi.org/10.2210/pdb1FUT/pdb> | Catalytic domain | 0.89 |
| I91 | PSMA5 | <https://doi.org/10.2210/pdb1FUT/pdb> | Catalytic domain | 0.86 |
| C97 | PSMB3 | <https://doi.org/10.2210/pdb1FUT/pdb> | Catalytic domain | 0.93 |
| I96 | PSMB3 | <https://doi.org/10.2210/pdb1FUT/pdb> | Catalytic domain | 0.9 |
| A98 | PSMB3 | <https://doi.org/10.2210/pdb1FUT/pdb> | Catalytic domain | 0.87 |
| G103 | PSMB3 | <https://doi.org/10.2210/pdb1FUT/pdb> | Catalytic domain | 0.85 |
| T102 | PSMB3 | <https://doi.org/10.2210/pdb1FUT/pdb> | Catalytic domain | 0.88 |
