# Supplementary material for: Exosomal gene-based predictive model and therapeutic target identification for Alzheimer’s disease: A bioinformatics analysis
Source: PLoS One. 2026 Jul 20;21(7):e0354014. doi: 10.1371/journal.pone.0354014 (PMC13384285; doi:10.1371/journal.pone.0354014)
Supplement: Attachment — Submitted filename: Response to Reviewers.docx [file pone.0354014.s018.docx]

To editor

Please ensure that you refer to Figure 2 in your text as, if accepted, production will need this reference to link the reader to the figure.

Thank you for your suggestion. We have enriched this section accordingly.

To reviewer 1:

1: 1. The title contains many concepts (exosomes, genetics, prediction, pharmacology, networks, docking), which makes it hard to understand the main focus.

Thank you for your guidance. We have revised the title of the manuscript.

2. The role of exosomes and their importance in Alzheimer’s disease is explained many times across the abstract, introduction, and discussion with little new information.

Thank you for your guidance. We have expanded and enriched this section in the manuscript.

3. There are many grammatical errors, awkward sentences, and inconsistent phrasing that reduce clarity and readability.

Thank you for your guidance. We have revised the manuscript for grammatical errors and awkward expressions.

4. Key steps (e.g., data normalization, batch correction, model validation strategy) are missing, making the study hard to reproduce.

Thank you for your guidance. We have enriched this section in the manuscript accordingly.

5. The four GEO datasets come from different sources, but there is no clear explanation of how batch effects or sample differences were handled.

As these four datasets were all derived from human brain tissue with uniform platforms, we have further described the standardization of samples in the Methods section and uploaded the relevant analysis code.

6. It is not well explained how exosomal genes were defined or filtered from GeneCards, and no confidence score or cutoff is mentioned.

Thank you for your guidance. We mainly searched the keyword "exosomes" in this database and selected genes with a score ≥ 2 as exosome-related genes.
7. The choice of LASSO, SVM, and random forest is not justified, and parameters used for these models are not reported.

In this study, three machine learning algorithms—LASSO regression, support vector machine (SVM) and random forest—were combined to screen the core characteristic exosome genes of Alzheimer’s disease (AD). This was not a single-algorithm screening, but a scientific selection based on the complementary technical characteristics of the three algorithms and the research requirements of AD gene screening. The screening logic and rationality are as follows:

8. The same datasets appear to be used for feature selection and model evaluation, which may inflate model performance.

Thank you for your guidance. We have expanded and enriched this section accordingly.

9. An AUC just above 0.70 indicates moderate accuracy, but the manuscript describes the model as ‘robust,’ which may be overstated.

Thank you for your guidance. We have revised this section in the original manuscript, emphasizing that our model is a reliable one with moderate accuracy.

10. The model is not validated on an external dataset, which limits its clinical reliability.

Thank you for your guidance. A significant limitation of this study lies in the lack of external validation in an independent cohort, which is influenced by the limited access to well-annotated gene expression datasets of Alzheimer's disease (AD) brain tissue. Due to ethical sampling requirements and practical collection challenges, brain tissue samples from AD patients are scarce. To compensate for this limitation, we employed multiple rigorous internal validation methods to verify the stability of the model. Future research will collaborate with multi-center clinical institutions to collect independent AD brain tissue samples for external validation, which will further verify the clinical applicability of the 5-gene biomarker.

11. The functional roles of CD44, CXCR4, TUBB, PSMA5, and PSMB3 in AD are not

deeply or critically analyzed.

CD44, a receptor for hyaluronic acid, is upregulated in patients with Alzheimer’s disease (AD) and exerts protective effects against AD by regulating V‑ATPase to ameliorate endolysosomal dysfunction and reduce protein toxicity and neurotoxicity [1]. In AD, the expression of CD44 and its splice variants (CD44V3, CD44V6, CD44V10) is significantly higher in the hippocampus of patients than in non‑AD controls. CD44S is mainly localized to neuritic plaques and astrocytes, while CD44V3, CD44V6, and CD44V10 are predominantly concentrated in neurons. Aβ peptides can induce the expression of CD44V6 and CD44V10, and CD44V10 is causally associated with Aβ‑induced neuronal toxicity and cell death. Inhibition of CD44V10 protects neurons, representing a promising novel neuroprotective therapeutic strategy for AD [2].

CXCR4, a highly conserved seven‑transmembrane G protein‑coupled receptor, forms a signaling axis with its specific ligand CXCL12 that plays a critical regulatory role in the pathological progression of AD. This pathway is involved not only in neurotransmission, synaptic plasticity, and neuroinflammation in the central nervous system but also mediates the regulation of multiple AD‑related downstream signaling pathways including MAPK, PI3K‑AKT, and NF‑κB via Gi proteins. Studies have shown that the expression of CXCL12/CXCR4 is significantly downregulated in the brains of AD patients and animal models, disrupting normal neuron‑glia communication and leading to synaptic dysfunction and impaired learning and memory. It is also closely associated with pathological alterations such as microglial activation, neuroinflammation, and excitotoxicity, mediating core processes including β‑amyloid deposition, Tau hyperphosphorylation, and cognitive decline. These findings suggest that the CXCL12/CXCR4 axis may serve as a potentially important target for early prediction, mechanistic research, and targeted therapy of AD [3].

TUBB (β‑tubulin), a key component of the cytoskeleton, has been identified as a core biomarker in urinary diagnostic models for the mild cognitive impairment (MCI) stage of AD and is also a critical hub gene linked to AD. Its protein structure and post‑translational modifications are significantly altered in the brain tissue of AD patients. By affecting microtubule assembly and stability, neuronal structural integrity, and axonal transport, TUBB contributes to neuronal damage and cognitive decline during AD pathogenesis. Characteristic changes in urinary TUBB are detectable as early as the MCI stage, indicating its potential noninvasive diagnostic value for the early identification of preclinical AD pathological changes [4].

PSMA5 is a key circadian rhythm‑related biomarker in AD. Its expression is decreased in Aβ‑induced neuronal injury, and it participates in AD pathogenesis by modulating the ubiquitin‑proteasome system, immune microenvironment, and circadian homeostasis [5].

In a Mendelian randomization study integrating brain proteomic and genetic data, PSMB3 showed no causal association with Alzheimer’s disease. Instead, it acts as a potential pathogenic target for amyotrophic lateral sclerosis and is involved in neuronal injury and degeneration by regulating proteasome function [6].

1.	Kim, S.H., et al., Endolysosomal impairment by binding of amyloid beta or MAPT/Tau to V-ATPase and rescue via the HYAL-CD44 axis in Alzheimer disease. Autophagy, 2023. 19(8): p. 2318-2337.

2.	Pinner, E., et al., CD44 Splice Variants as Potential Players in Alzheimer's Disease Pathology. J Alzheimers Dis, 2017. 58(4): p. 1137-1149.

3.	Li, H. and R. Wang, A focus on CXCR4 in Alzheimer's disease. Brain Circ, 2017. 3(4): p. 199-203.

4.	Wang, Y., et al., Identification of novel diagnostic panel for mild cognitive impairment and Alzheimer's disease: findings based on urine proteomics and machine learning. Alzheimers Res Ther, 2023. 15(1): p. 191.

5.	He, H., et al., Combined analysis of single-cell and bulk RNA sequencing reveals the expression patterns of circadian rhythm disruption in the immune microenvironment of Alzheimer's disease. Front Immunol, 2023. 14: p. 1182307.

6.	Ge, Y.J., et al., Prioritization of Drug Targets for Neurodegenerative Diseases by Integrating Genetic and Proteomic Data From Brain and Blood. Biol Psychiatry, 2023. 93(9): p. 770-779.

12. Some identified compounds (e.g., phencyclidine, thioridazine) are neurotoxic or clinically inappropriate, but this is not discussed.

We have incorporated this discussion into the manuscript.

Oxazolone, whose lead candidate 15E shows high specificity for Aβ oligomer/fibril formation, enables early diagnosis of Alzheimer's disease. It can precisely release valproic acid in the Alzheimer's disease microenvironment, effectively inhibiting/degrading Aβ fibril formation, disrupting fibril structure, and alleviating Aβ-induced neurotoxicity. This oxazolone-based photosensitive cage integrates diagnostic and therapeutic functions.

In patients with Alzheimer's disease, the phencyclidine-binding channel sites of NMDA receptors (labeled with [³H]MK-801) are the most significantly impaired, with a mean 34% reduction in binding capacity in the CA1 stratum pyramidale of the hippocampus; this decrease is most likely due to reduced receptor density and is uncorrelated with neuronal loss, plaque and tangle density.

As a typical antipsychotic, thioridazine can inhibit psychotic symptoms in Alzheimer's disease patients by regulating the serotonin and dopamine pathways, thereby indirectly alleviating skin-picking self-injurious behaviors induced by such symptoms; although a single case has shown it can achieve complete remission of these symptoms, the drug lacks clinical trial validation, may increase the risk of cerebrovascular diseases and cause various adverse reactions in patients, and has no direct effect on the core pathology of Alzheimer's disease.

13. Docking targets are listed only as amino acid residues without specifying the protein structures or binding context.

We appreciate the reviewer’s comment and apologize for the omission. The protein structures and binding contexts for the docking amino acid residues have been added to the revised manuscript and a supplementary table (Table S1). These residues map to the core proteins of the 5-gene signature (CD44/CXCR4/TUBB/PSMA5/PSMB3; PDB IDs provided) and are localized in their functional/conserved domains (extracellular/catalytic/core), with full details in the supplementary materials.

14. The study is entirely computational, and no in vitro, in vivo, or clinical validation is provided.

Thank you for your guidance. A significant limitation of this study lies in the lack of external validation in an independent cohort, which is influenced by the limited access to well-annotated gene expression datasets of Alzheimer's disease (AD) brain tissue. Due to ethical sampling requirements and practical collection challenges, brain tissue samples from AD patients are scarce. To compensate for this limitation, we employed multiple rigorous internal validation methods to verify the stability of the model. Future research will collaborate with multi-center clinical institutions to collect independent AD brain tissue samples for external validation, which will further verify the clinical applicability of the 5-gene biomarker.

15. Many figures are cited, but their key messages are not clearly described in the text.

We appreciate the reviewer’s comment and apologize for the unclear description of figure key messages in the original text. We have comprehensively revised the Results section, and added specific, corresponding descriptions of the core findings and biological implications for each cited figure (1–9) at the citation position, tightly integrating figure results with text narrative to enhance the interpretability and logical coherence of the study.

16. Correlations with immune cells are reported, but their biological meaning in AD pathology is not explored in depth.

We appreciate the reviewer’s comment. We acknowledge the insufficient exploration of the biological significance of the immune cell correlation in AD pathogenesis, and have added a dedicated section in the revised Discussion to elaborate on the mechanistic link between the 5-gene signature, immune cell regulation (B cells, CD4+/CD8+ T cells, MDSCs) and key AD pathological processes (neuroinflammation, Aβ deposition, tau hyperphosphorylation), combining exosome’s intercellular communication function to clarify its specific biological implications.

17. The discussion mainly restates findings rather than critically interpreting their significance or limitations.

We appreciate the reviewers' constructive comments and fully acknowledge the shortcomings of the original discussion section — it overly emphasized restating results and lacked in-depth critical interpretation of their significance and limitations. We have comprehensively revised this section in the revised manuscript, adding research related to the five genes in AD and discussing their significance.

18. Claims such as ‘paradigm shift’ and ‘precision targeting’ are too strong given the lack of experimental validation.

We appreciate the reviewer’s comment and fully acknowledge that paradigm shift and precision targeting are overstated due to the lack of experimental validation. These terms have been completely removed from the revised manuscript, and all relevant descriptions have been replaced with cautious, objective wording that matches the exploratory bioinformatic nature of our study, with no overinterpretation of the findings.

19. Drug names, gene names, and abbreviations are inconsistently written (e.g., PHENCYYCLIDINE, spacing errors, punctuation issues).

We thank the reviewer for this careful comment. We have carefully checked and unified the writing format of all drug names, gene names, and abbreviations throughout the manuscript, including correcting spelling errors (e.g., phencyclidine), inconsistent capitalization, spacing mistakes, and punctuation issues. All terms are now presented in a consistent and standardized manner.

20. Potential risks, translational barriers, and clinical feasibility of using exosomal gene models are not discussed.

We sincerely appreciate the reviewer’s valuable comment. We acknowledge that the potential risks, translational barriers, and clinical feasibility of using the exosome gene model for AD prediction were not sufficiently discussed in the original manuscript. These critical points have been comprehensively added and elaborated in the revised Discussion section, including safety considerations, technical challenges in clinical application, ethical constraints, and practical obstacles in clinical translation. We have also provided objective and balanced interpretations of the clinical applicability of the current model, fully considering its advantages and limitations.

To reviewer 2：

Reviewer #2: Overall, the study addresses an interesting and relevant topic. With clearer methodological descriptions and more cautious interpretation of the findings, the manuscript could be significantly strengthened.

Major Comments
• The manuscript refers to “exosomal genes,” but the datasets used are bulk transcriptomic datasets rather than samples derived from isolated exosomes. It would help if the authors clarified how these genes were defined as exosome-related and provided stronger justification for linking them to exosomal biology in Alzheimer’s disease.

Thank you for your valuable comment. We clarify that the bulk transcriptomic datasets used in this study were from the GEO database, with all samples being human brain tissue of AD patients and normal controls. We defined exosome-related genes via dual validation: intersecting AD differential genes from the above brain tissue bulk data with exosome-related genes (relevance score ≥ 2) from the GeneCards database. Moreover, published studies have confirmed that our identified 5 core genes are enriched in exosomes secreted by brain CNS cells and participate in AD exosomal biological processes. Relevant screening details and literature citations have been supplemented in the revised manuscript (marked in red). We thank you again for your comment that improved the rigor of our study.

• Some important methodological details are missing. In particular, the manuscript should clearly describe how batch effects between the GEO datasets were handled and how the datasets were integrated for analysis.

Thank you for your valuable comment. We apologize for the missing methodological details and have supplemented the complete information on batch effect correction and dataset integration in the Methods section of the revised manuscript (marked in red). Specifically, we first removed batch effects among the four GEO datasets using the sva package in R software to eliminate inter-dataset systematic biases; the corrected data were then integrated for subsequent differential expression and joint analysis, with PCA plots before and after batch correction (Figure 1) validating the correction effect intuitively. In addition, the code for data normalization analysis has been uploaded as a supplementary file to the submission system. We thank you again for your comment that improved the methodological rigor of our study.

• The predictive model shows AUC values above 0.70, which is encouraging. However, the manuscript does not clearly state whether cross-validation or an independent validation dataset was used. Adding this information would strengthen confidence in the model and reduce the possibility of overfitting.

Thank you for your comment. We have performed both **independent validation (Figure 6D)** and **cross-validation (Figure 6E)** to evaluate the model and avoid overfitting. Detailed descriptions have been added in the Methods and Results sections (marked in red).

• The molecular docking section needs more clarity. It would be helpful to include the docking software used, the protein structures selected, and the binding affinity or docking score values so that the analysis can be better understood and reproduced.

Thank you for your comment. We have supplemented the docking website in the Methods section, and listed detailed in Supplementary Table S1

• A few biological interpretations need correction or moderation. For example, CD44 and CXCR4 are described as transcription factors, while they are actually membrane receptors. Adjusting these descriptions would improve the scientific accuracy of the manuscript.

Thank you for this important correction. We sincerely apologize for the inaccurate description.CD44 and CXCR4 are membrane receptors, not transcription factors. We have revised all relevant statements in the manuscript (marked in red) to ensure scientific accuracy.

Minor Comments
• There are a few typographical and formatting errors throughout the manuscript that should be corrected during revision.

Thank you for your guidance. We have revised the relevant content accordingly.

• Some figure legends could be expanded slightly to help readers understand what is being shown and how the analyses were performed.

Thank you for your comment. We have expanded and supplemented the figure legends to clarify the content

• The discussion could benefit from including a few more recent references related to exosome-based biomarkers in Alzheimer’s disease.

Thank you for your suggestion. We have enriched this section accordingly.

• A small amount of language polishing and tightening of sentences would improve readability.

We have revised and improved this section accordingly.
