# Supplementary material for: Exosomal gene-based predictive model and therapeutic target identification for Alzheimer’s disease: A bioinformatics analysis
Source: PLoS One. 2026 Jul 20;21(7):e0354014. doi: 10.1371/journal.pone.0354014 (PMC13384285; doi:10.1371/journal.pone.0354014)
Supplement: Attachment — Submitted filename: Response_to_Reviewers_auresp_2.docx [file pone.0354014.s019.docx]

Dear Editor and Reviewers,

We greatly appreciate the editors and reviewers for their valuable and constructive comments, which have significantly improved the quality of our manuscript. We have carefully addressed all concerns, and detailed point-by-point responses are provided below.

Response to Reviewer 1

We sincerely appreciate your rigorous and thoughtful review. We fully acknowledge that our previous responses were relatively general and lacked specific descriptions of the exact revisions made in the manuscript. To fully address your concerns regarding methodological transparency, reproducibility, validation rigor, and consistency, we have now completely and thoroughly revised the manuscript, with all changes clearly marked in the tracked version. We have supplemented every key step with explicit, step-by-step details to ensure full clarity and scientific rigor. Our point-by-point response is as follows:

1. The title contained too many concepts and was difficult to follow.

Response:

We have simplified and refined the title to highlight the core focus.

Revised title:

Exosomal Gene-Based Predictive Model and Therapeutic Target Identification for Alzheimer's Disease: A Bioinformatics Analysis

2. Repetitive descriptions of exosomes across sections.

Response:

We have streamlined redundant descriptions and retained only essential background information. Mechanistic insights and translational implications are concentrated in the Discussion to improve logical flow and readability.

3. Grammatical errors, awkward sentences, and inconsistent phrasing.

Response:

The entire manuscript has been professionally edited to correct grammatical errors, optimize sentence structure, and ensure consistent terminology throughout.

4. Key methodological steps missing (normalization, batch correction, validation); study not reproducible.

Response:

We have added complete, explicit, step-by-step protocols in the Methods section, including:

Data normalization (log₂ transformation + between-array normalization)

Batch effect correction using the ComBat algorithm

Full model validation (10-fold cross-validation, calibration, DCA)

All analysis code is provided as supplementary files to ensure full reproducibility.

5. Four GEO datasets from different sources; no clear batch effect handling.

Response:

We have added clear descriptions: batch effects across datasets were eliminated using the ComBat function from the sva package. PCA plots before and after correction are presented to confirm successful removal of batch bias. Corresponding code is available in SVA.txt.

6. Unclear definition/filtering of exosomal genes from GeneCards; no cutoff stated.

Response:

Exosome-related genes were retrieved from GeneCards using the keyword “exosomes” with a strict cutoff of relevance score ≥ 2. This criterion is clearly stated in the Methods.

7. Choice of LASSO, SVM, random forest not justified; parameters not reported.

Response:

We have added detailed justification and complete parameters:

LASSO: feature dimension reduction; 10-fold cross-validation; λ selected by minimum error

SVM-RFE: identify optimal gene sets with minimum cross-validation error

Random Forest: ntree = 500; gene importance scored by MeanDecreaseGini

All justifications and code files are clearly reported.

8. Same dataset used for feature selection and model evaluation, which may inflate performance.

Response:

We have adopted 10-fold cross-validation and independent train-test splitting to completely separate feature selection and model evaluation, eliminating data leakage and overfitting. All results were updated accordingly.

9. AUC > 0.7 described as “robust”; overstated.

Response:

We have removed “robust” and revised to moderate and stable predictive performance (AUC > 0.70), consistent with the exploratory bioinformatic nature of this study.

10. No external validation; limited clinical reliability.

Response:

We fully acknowledge this limitation. Owing to the scarcity of well-annotated AD brain tissue datasets under ethical constraints, we performed rigorous internal validation including bootstrap resampling, 10-fold cross-validation, calibration, and DCA. Multi-center external validation will be conducted in future studies.

11. Insufficient mechanistic analysis of CD44, CXCR4, TUBB, PSMA5, PSMB3 in AD.

Response:

We have added a comprehensive mechanistic discussion for each gene in AD pathogenesis, including their roles in neuroinflammation, Aβ clearance, tau pathology, cytoskeleton stability, proteasome function, and circadian regulation, supported by relevant published literature.

12. Some identified compounds (phencyclidine, thioridazine) are neurotoxic or clinically inappropriate; not discussed.

Response:

We have added critical safety and clinical suitability discussions. These compounds are included only as molecular probes, not for direct clinical use, and their potential risks are clearly addressed.

13. Docking targets listed only as residues without protein structure or binding context.

Response:

We have supplemented corresponding PDB IDs, functional domains, and binding environments for all docking residues. Details are included in the manuscript and supplementary materials.

14. The study is entirely computational; no experimental validation.

Response:

We recognize this as a major limitation and clearly state it in the manuscript. This study provides exploratory computational predictions; experimental validation will be performed in future work.

15. Figures cited but key messages not clearly described in the text.

Response:

We have added clear biological interpretations for all figures (1–9) in the Results section. Each panel is explained with its core finding and implication.

16. Immune cell correlations reported but biological meaning in AD not explored.

Response:

We have added detailed discussion linking the 5-gene signature to immune cells (B cells, CD4+/CD8+ T cells, MDSCs) and AD neuroinflammation, amyloid deposition, and tau hyperphosphorylation.

17. Discussion mainly restates results rather than providing critical interpretation.

Response:

We have completely rewritten the Discussion to emphasize mechanistic insights, translational value, literature comparison, and in-depth limitations, rather than only summarizing results.

18. Overstated claims such as “paradigm shift” and “precision targeting.”

Response:

All exaggerated statements have been removed. Conclusions are now cautious, objective, and consistent with a computational bioinformatics study.

19. Inconsistent formatting of drug/gene/abbreviation names (e.g., PHENCYYCLIDINE).

Response:

All gene symbols, drug names, abbreviations, and spellings have been carefully checked and standardized. “Phencyclidine” is now correctly used throughout.

20. Potential risks, translational barriers, and clinical feasibility not discussed.

Response:

We have added a dedicated section discussing clinical translation challenges: exosome isolation standardization, cost, ethical constraints, non-specific signals, and implementation hurdles.

We greatly appreciate your valuable and professional guidance, which has significantly improved the quality and rigor of our manuscript. We sincerely hope these detailed, transparent, and comprehensive revisions fully address all remaining concerns.

Response to Reviewer 2

We greatly appreciate your positive and constructive feedback. We are delighted that our revisions have met your approval. As suggested, we have:

Further polished the English language and fluency of the entire manuscript.

Corrected all minor formatting inconsistencies throughout the text.

Briefly clarified the details of molecular docking (including binding residues and structural context) in the main text.

Thank you again for your valuable comments, which have greatly improved the presentation of our work.
