# Supplementary material for: Exosomal gene-based predictive model and therapeutic target identification for Alzheimer’s disease: A bioinformatics analysis
Source: PLoS One. 2026 Jul 20;21(7):e0354014. doi: 10.1371/journal.pone.0354014 (PMC13384285; doi:10.1371/journal.pone.0354014)
Supplement: Peer review history

# Peer review: Decision Letter 0

**DOI:** 10.1371/journal.pone.0354014.r001

**Authors:** Amr Ahmed El-Arabey

13 Mar 2026

PONE-D-25-47921

Exosomal Genetic Signature-Based Predictive Modeling for Alzheimer's Disease: Integrated Pharmacological Network Analysis and Therapeutic Target Discovery

PLOS One

Dear Dr. Ma,

Thank you for submitting your manuscript to PLOS ONE. After careful consideration, we feel that it has merit but does not fully meet PLOS ONE’s publication criteria as it currently stands. Therefore, we invite you to submit a revised version of the manuscript that addresses the points raised during the review process.

Please submit your revised manuscript by Apr 27 2026 11:59PM. If you will need more time than this to complete your revisions, please reply to this message or contact the journal office at plosone@plos.org. When you're ready to submit your revision, log on to https://www.editorialmanager.com/pone/ and select the 'Submissions Needing Revision' folder to locate your manuscript file.

Please include the following items when submitting your revised manuscript:

- A letter that responds to each point raised by the academic editor and reviewer(s). You should upload this letter as a separate file labeled 'Response to Reviewers'.
- A marked-up copy of your manuscript that highlights changes made to the original version. You should upload this as a separate file labeled 'Revised Manuscript with Track Changes'.
- An unmarked version of your revised paper without tracked changes. You should upload this as a separate file labeled 'Manuscript'.

If you would like to make changes to your financial disclosure, please include your updated statement in your cover letter. Guidelines for resubmitting your figure files are available below the reviewer comments at the end of this letter.

If applicable, we recommend that you deposit your laboratory protocols in protocols.io to enhance the reproducibility of your results. Protocols.io assigns your protocol its own identifier (DOI) so that it can be cited independently in the future. For instructions see: https://journals.plos.org/plosone/s/submission-guidelines#loc-laboratory-protocols. Additionally, PLOS ONE offers an option for publishing peer-reviewed Lab Protocol articles, which describe protocols hosted on protocols.io. Read more information on sharing protocols at https://plos.org/protocols?utm_medium=editorial-email&utm_source=authorletters&utm_campaign=protocols.

We look forward to receiving your revised manuscript.

Kind regards,

Amr Ahmed El-Arabey

Academic Editor

PLOS One

Journal Requirements:

When submitting your revision, we need you to address these additional requirements.

1. Please ensure that your manuscript meets PLOS ONE's style requirements, including those for file naming. The PLOS ONE style templates can be found at

https://journals.plos.org/plosone/s/file?id=wjVg/PLOSOne_formatting_sample_main_body.pdf and

https://journals.plos.org/plosone/s/file?id=ba62/PLOSOne_formatting_sample_title_authors_affiliations.pdf

2. Please note that PLOS One has specific guidelines on code sharing for submissions in which author-generated code underpins the findings in the manuscript. In these cases, we expect all author-generated code to be made available without restrictions upon publication of the work. Please review our guidelines at https://journals.plos.org/plosone/s/materials-and-software-sharing#loc-sharing-code and ensure that your code is shared in a way that follows best practice and facilitates reproducibility and reuse.

3. PLOS requires an ORCID iD for the corresponding author in Editorial Manager on papers submitted after December 6th, 2016. Please ensure that you have an ORCID iD and that it is validated in Editorial Manager. To do this, go to ‘Update my Information’ (in the upper left-hand corner of the main menu), and click on the Fetch/Validate link next to the ORCID field. This will take you to the ORCID site and allow you to create a new iD or authenticate a pre-existing iD in Editorial Manager.

4. Thank you for uploading your study's underlying data set. Unfortunately, the repository you have noted in your Data Availability statement does not qualify as an acceptable data repository according to PLOS's standards.

At this time, please upload the minimal data set necessary to replicate your study's findings to a stable, public repository (such as figshare or Dryad) and provide us with the relevant URLs, DOIs, or accession numbers that may be used to access these data. For a list of recommended repositories and additional information on PLOS standards for data deposition, please see https://journals.plos.org/plosone/s/recommended-repositories.

5. Please ensure that you refer to Figure 2 in your text as, if accepted, production will need this reference to link the reader to the figure.

6. If the reviewer comments include a recommendation to cite specific previously published works, please review and evaluate these publications to determine whether they are relevant and should be cited. There is no requirement to cite these works unless the editor has indicated otherwise.

[Note: HTML markup is below. Please do not edit.]

Reviewers' comments:

Reviewer's Responses to Questions

**Comments to the Author**

1. Is the manuscript technically sound, and do the data support the conclusions?

The manuscript must describe a technically sound piece of scientific research with data that supports the conclusions. Experiments must have been conducted rigorously, with appropriate controls, replication, and sample sizes. The conclusions must be drawn appropriately based on the data presented.

Reviewer #1: Partly

Reviewer #2: Yes

**********

2. Has the statistical analysis been performed appropriately and rigorously?

Reviewer #1: Yes

Reviewer #2: No

**********

3. Have the authors made all data underlying the findings in their manuscript fully available?

The PLOS Data policy requires authors to make all data underlying the findings described in their manuscript fully available without restriction, with rare exception (please refer to the Data Availability Statement in the manuscript PDF file). The data should be provided as part of the manuscript or its supporting information, or deposited to a public repository. For example, in addition to summary statistics, the data points behind means, medians and variance measures should be available. If there are restrictions on publicly sharing data—e.g. participant privacy or use of data from a third party—those must be specified.

Reviewer #1: Yes

Reviewer #2: No

**********

4. Is the manuscript presented in an intelligible fashion and written in standard English?

PLOS ONE does not copyedit accepted manuscripts, so the language in submitted articles must be clear, correct, and unambiguous. Any typographical or grammatical errors should be corrected at revision, so please note any specific errors here.

Reviewer #1: No

Reviewer #2: Yes

**********

5. Review Comments to the Author

Please use the space provided to explain your answers to the questions above. You may also include additional comments for the author, including concerns about dual publication, research ethics, or publication ethics. (Please upload your review as an attachment if it exceeds 20,000 characters)

Reviewer #1: 1. The title contains many concepts (exosomes, genetics, prediction, pharmacology, networks, docking), which makes it hard to understand the main focus.

2. The role of exosomes and their importance in Alzheimer’s disease is explained many times across the abstract, introduction, and discussion with little new information.

3. There are many grammatical errors, awkward sentences, and inconsistent phrasing that reduce clarity and readability.

4. Key steps (e.g., data normalization, batch correction, model validation strategy) are missing, making the study hard to reproduce.

5. The four GEO datasets come from different sources, but there is no clear explanation of how batch effects or sample differences were handled.

6. It is not well explained how exosomal genes were defined or filtered from GeneCards, and no confidence score or cutoff is mentioned.

7. The choice of LASSO, SVM, and random forest is not justified, and parameters used for these models are not reported.

8. The same datasets appear to be used for feature selection and model evaluation, which may inflate model performance.

9. An AUC just above 0.70 indicates moderate accuracy, but the manuscript describes the model as ‘robust,’ which may be overstated.

10. The model is not validated on an external dataset, which limits its clinical reliability.

11. The functional roles of CD44, CXCR4, TUBB, PSMA5, and PSMB3 in AD are not deeply or critically analyzed.

12. Some identified compounds (e.g., phencyclidine, thioridazine) are neurotoxic or clinically inappropriate, but this is not discussed.

13. Docking targets are listed only as amino acid residues without specifying the protein structures or binding context.

14. The study is entirely computational, and no in vitro, in vivo, or clinical validation is provided.

15. Many figures are cited, but their key messages are not clearly described in the text.

16. Correlations with immune cells are reported, but their biological meaning in AD pathology is not explored in depth.

17. The discussion mainly restates findings rather than critically interpreting their significance or limitations.

18. Claims such as ‘paradigm shift’ and ‘precision targeting’ are too strong given the lack of experimental validation.

19. Drug names, gene names, and abbreviations are inconsistently written (e.g., PHENCYYCLIDINE, spacing errors, punctuation issues).

20. Potential risks, translational barriers, and clinical feasibility of using exosomal gene models are not discussed.

Reviewer #2: Overall, the study addresses an interesting and relevant topic. With clearer methodological descriptions and more cautious interpretation of the findings, the manuscript could be significantly strengthened.

Major Comments

• The manuscript refers to “exosomal genes,” but the datasets used are bulk transcriptomic datasets rather than samples derived from isolated exosomes. It would help if the authors clarified how these genes were defined as exosome-related and provided stronger justification for linking them to exosomal biology in Alzheimer’s disease.

• Some important methodological details are missing. In particular, the manuscript should clearly describe how batch effects between the GEO datasets were handled and how the datasets were integrated for analysis.

• The predictive model shows AUC values above 0.70, which is encouraging. However, the manuscript does not clearly state whether cross-validation or an independent validation dataset was used. Adding this information would strengthen confidence in the model and reduce the possibility of overfitting.

• The molecular docking section needs more clarity. It would be helpful to include the docking software used, the protein structures selected, and the binding affinity or docking score values so that the analysis can be better understood and reproduced.

• A few biological interpretations need correction or moderation. For example, CD44 and CXCR4 are described as transcription factors, while they are actually membrane receptors. Adjusting these descriptions would improve the scientific accuracy of the manuscript.

Minor Comments

• There are a few typographical and formatting errors throughout the manuscript that should be corrected during revision.

• Some figure legends could be expanded slightly to help readers understand what is being shown and how the analyses were performed.

• The discussion could benefit from including a few more recent references related to exosome-based biomarkers in Alzheimer’s disease.

• A small amount of language polishing and tightening of sentences would improve readability.

**********

6. PLOS authors have the option to publish the peer review history of their article (what does this mean?). If published, this will include your full peer review and any attached files.

If you choose “no”, your identity will remain anonymous but your review may still be made public.

**Do you want your identity to be public for this peer review?**  For information about this choice, including consent withdrawal, please see our Privacy Policy.

Reviewer #1: No

Reviewer #2: No

**********

[NOTE: If reviewer comments were submitted as an attachment file, they will be attached to this email and accessible via the submission site. Please log into your account, locate the manuscript record, and check for the action link "View Attachments". If this link does not appear, there are no attachment files.]

To ensure your figures meet our technical requirements, please review our figure guidelines: https://journals.plos.org/plosone/s/figures

You may also use PLOS’s free figure tool, NAAS, to help you prepare publication quality figures: https://journals.plos.org/plosone/s/figures#loc-tools-for-figure-preparation.

NAAS will assess whether your figures meet our technical requirements by comparing each figure against our figure specifications.

# Author comment: Author response to Decision Letter 1

**DOI:** 10.1371/journal.pone.0354014.r002

9 Apr 2026

To editor

Please ensure that you refer to Figure 2 in your text as, if accepted, production will need this reference to link the reader to the figure.

Thank you for your suggestion. We have enriched this section accordingly.

To reviewer 1:

1: 1. The title contains many concepts (exosomes, genetics, prediction, pharmacology, networks, docking), which makes it hard to understand the main focus.

Thank you for your guidance. We have revised the title of the manuscript.

2. The role of exosomes and their importance in Alzheimer’s disease is explained many times across the abstract, introduction, and discussion with little new information.

Thank you for your guidance. We have expanded and enriched this section in the manuscript.

3. There are many grammatical errors, awkward sentences, and inconsistent phrasing that reduce clarity and readability.

Thank you for your guidance. We have revised the manuscript for grammatical errors and awkward expressions.

4. Key steps (e.g., data normalization, batch correction, model validation strategy) are missing, making the study hard to reproduce.

Thank you for your guidance. We have enriched this section in the manuscript accordingly.

5. The four GEO datasets come from different sources, but there is no clear explanation of how batch effects or sample differences were handled.

As these four datasets were all derived from human brain tissue with uniform platforms, we have further described the standardization of samples in the Methods section and uploaded the relevant analysis code.

6. It is not well explained how exosomal genes were defined or filtered from GeneCards, and no confidence score or cutoff is mentioned.

Thank you for your guidance. We mainly searched the keyword "exosomes" in this database and selected genes with a score ≥ 2 as exosome-related genes.

7. The choice of LASSO, SVM, and random forest is not justified, and parameters used for these models are not reported.

In this study, three machine learning algorithms—LASSO regression, support vector machine (SVM) and random forest—were combined to screen the core characteristic exosome genes of Alzheimer’s disease (AD). This was not a single-algorithm screening, but a scientific selection based on the complementary technical characteristics of the three algorithms and the research requirements of AD gene screening. The screening logic and rationality are as follows:

8. The same datasets appear to be used for feature selection and model evaluation, which may inflate model performance.

Thank you for your guidance. We have expanded and enriched this section accordingly.

9. An AUC just above 0.70 indicates moderate accuracy, but the manuscript describes the model as ‘robust,’ which may be overstated.

Thank you for your guidance. We have revised this section in the original manuscript, emphasizing that our model is a reliable one with moderate accuracy.

10. The model is not validated on an external dataset, which limits its clinical reliability.

Thank you for your guidance. A significant limitation of this study lies in the lack of external validation in an independent cohort, which is influenced by the limited access to well-annotated gene expression datasets of Alzheimer's disease (AD) brain tissue. Due to ethical sampling requirements and practical collection challenges, brain tissue samples from AD patients are scarce. To compensate for this limitation, we employed multiple rigorous internal validation methods to verify the stability of the model. Future research will collaborate with multi-center clinical institutions to collect independent AD brain tissue samples for external validation, which will further verify the clinical applicability of the 5-gene biomarker.

11. The functional roles of CD44, CXCR4, TUBB, PSMA5, and PSMB3 in AD are not

deeply or critically analyzed.

CD44, a receptor for hyaluronic acid, is upregulated in patients with Alzheimer’s disease (AD) and exerts protective effects against AD by regulating V ATPase to ameliorate endolysosomal dysfunction and reduce protein toxicity and neurotoxicity [1]. In AD, the expression of CD44 and its splice variants (CD44V3, CD44V6, CD44V10) is significantly higher in the hippocampus of patients than in non AD controls. CD44S is mainly localized to neuritic plaques and astrocytes, while CD44V3, CD44V6, and CD44V10 are predominantly concentrated in neurons. Aβ peptides can induce the expression of CD44V6 and CD44V10, and CD44V10 is causally associated with Aβ induced neuronal toxicity and cell death. Inhibition of CD44V10 protects neurons, representing a promising novel neuroprotective therapeutic strategy for AD [2].

CXCR4, a highly conserved seven transmembrane G protein coupled receptor, forms a signaling axis with its specific ligand CXCL12 that plays a critical regulatory role in the pathological progression of AD. This pathway is involved not only in neurotransmission, synaptic plasticity, and neuroinflammation in the central nervous system but also mediates the regulation of multiple AD related downstream signaling pathways including MAPK, PI3K AKT, and NF κB via Gi proteins. Studies have shown that the expression of CXCL12/CXCR4 is significantly downregulated in the brains of AD patients and animal models, disrupting normal neuron glia communication and leading to synaptic dysfunction and impaired learning and memory. It is also closely associated with pathological alterations such as microglial activation, neuroinflammation, and excitotoxicity, mediating core processes including β amyloid deposition, Tau hyperphosphorylation, and cognitive decline. These findings suggest that the CXCL12/CXCR4 axis may serve as a potentially important target for early prediction, mechanistic research, and targeted therapy of AD [3].

TUBB (β tubulin), a key component of the cytoskeleton, has been identified as a core biomarker in urinary diagnostic models for the mild cognitive impairment (MCI) stage of AD and is also a critical hub gene linked to AD. Its protein structure and post translational modifications are significantly altered in the brain tissue of AD patients. By affecting microtubule assembly and stability, neuronal structural integrity, and axonal transport, TUBB contributes to neuronal damage and cognitive decline during AD pathogenesis. Characteristic changes in urinary TUBB are detectable as early as the MCI stage, indicating its potential noninvasive diagnostic value for the early identification of preclinical AD pathological changes [4].

PSMA5 is a key circadian rhythm related biomarker in AD. Its expression is decreased in Aβ induced neuronal injury, and it participates in AD pathogenesis by modulating the ubiquitin proteasome system, immune microenvironment, and circadian homeostasis [5].

In a Mendelian randomization study integrating brain proteomic and genetic data, PSMB3 showed no causal association with Alzheimer’s disease. Instead, it acts as a potential pathogenic target for amyotrophic lateral sclerosis and is involved in neuronal injury and degeneration by regulating proteasome function [6].

1. Kim, S.H., et al., Endolysosomal impairment by binding of amyloid beta or MAPT/Tau to V-ATPase and rescue via the HYAL-CD44 axis in Alzheimer disease. Autophagy, 2023. 19(8): p. 2318-2337.

2. Pinner, E., et al., CD44 Splice Variants as Potential Players in Alzheimer's Disease Pathology. J Alzheimers Dis, 2017. 58(4): p. 1137-1149.

3. Li, H. and R. Wang, A focus on CXCR4 in Alzheimer's disease. Brain Circ, 2017. 3(4): p. 199-203.

4. Wang, Y., et al., Identification of novel diagnostic panel for mild cognitive impairment and Alzheimer's disease: findings based on urine proteomics and machine learning. Alzheimers Res Ther, 2023. 15(1): p. 191.

5. He, H., et al., Combined analysis of single-cell and bulk RNA sequencing reveals the expression patterns of circadian rhythm disruption in the immune microenvironment of Alzheimer's disease. Front Immunol, 2023. 14: p. 1182307.

6. Ge, Y.J., et al., Prioritization of Drug Targets for Neurodegenerative Diseases by Integrating Genetic and Proteomic Data From Brain and Blood. Biol Psychiatry, 2023. 93(9): p. 770-779.

12. Some identified compounds (e.g., phencyclidine, thioridazine) are neurotoxic or clinically inappropriate, but this is not discussed.

We have incorporated this discussion into the manuscript.

Oxazolone, whose lead candidate 15E shows high specificity for Aβ oligomer/fibril formation, enables early diagnosis of Alzheimer's disease. It can precisely release valproic acid in the Alzheimer's disease microenvironment, effectively inhibiting/degrading Aβ fibril formation, disrupting fibril structure, and alleviating Aβ-induced neurotoxicity. This oxazolone-based photosensitive cage integrates diagnostic and therapeutic functions.

In patients with Alzheimer's disease, the phencyclidine-binding channel sites of NMDA receptors (labeled with [³H]MK-801) are the most significantly impaired, with a mean 34% reduction in binding capacity in the CA1 stratum pyramidale of the hippocampus; this decrease is most likely due to reduced receptor density and is uncorrelated with neuronal loss, plaque and tangle density.

As a typical antipsychotic, thioridazine can inhibit psychotic symptoms in Alzheimer's disease patients by regulating the serotonin and dopamine pathways, thereby indirectly alleviating skin-picking self-injurious behaviors induced by such symptoms; although a single case has shown it can achieve complete remission of these symptoms, the drug lacks clinical trial validation, may increase the risk of cerebrovascular diseases and cause various adverse reactions in patients, and has no direct effect on the core pathology of Alzheimer's disease.

13. Docking targets are listed only as amino acid residues without specifying the protein structures or binding context.

We appreciate the reviewer’s comment and apologize for the omission. The protein structures and binding contexts for the docking amino acid residues have been added to the revised manuscript and a supplementary table (Table S1). These residues map to the core proteins of the 5-gene signature (CD44/CXCR4/TUBB/PSMA5/PSMB3; PDB IDs provided) and are localized in their functional/conserved domains (extracellular/catalytic/core), with full details in the supplementary materials.

14. The study is entirely computational, and no in vitro, in vivo, or clinical validation is provided.

Thank you for your guidance. A significant limitation of this study lies in the lack of external validation in an independent cohort, which is influenced by the limited access to well-annotated gene expression datasets of Alzheimer's disease (AD) brain tissue. Due to ethical sampling requirements and practical collection challenges, brain tissue samples from AD patients are scarce. To compensate for this limitation, we employed multiple rigorous internal validation methods to verify the stability of the model. Future research will collaborate with multi-center clinical institutions to collect independent AD brain tissue samples for external validation, which will further verify the clinical applicability of the 5-gene biomarker.

15. Many figures are cited, but their key messages are not clearly described in the text.

We appreciate the reviewer’s comment and apologize for the unclear description of figure key messages in the original text. We have comprehensively revised the Results section, and added specific, corresponding descriptions of the core findings and biological implications for each cited figure (1–9) at the citation position, tightly integrating figure results with text narrative to enhance the interpretability and logical coherence of the study.

16. Correlations with immune cells are reported, but their biological meaning in AD pathology is not explored in depth.

We appreciate the reviewer’s comment. We acknowledge the insufficient exploration of the biological significance of the immune cell correlation in AD pathogenesis, and have added a dedicated section in the revised Discussion to elaborate on the mechanistic link between the 5-gene signature, immune cell regulation (B cells, CD4+/CD8+ T cells, MDSCs) and key AD pathological processes (neuroinflammation, Aβ deposition, tau hyperphosphorylation), combining exosome’s intercellular communication function to clarify its specific biological implications.

17. The discussion mainly restates findings rather than critically interpreting their significance or limitations.

We appreciate the reviewers' constructive comments and fully acknowledge the shortcomings of the original discussion section — it overly emphasized restating results and lacked in-depth critical interpretation of their significance and limitations. We have comprehensively revised this section in the revised manuscript, adding research related to the five genes in AD and discussing their significance.

18. Claims such as ‘paradigm shift’ and ‘precision targeting’ are too strong given the lack of experimental validation.

We appreciate the reviewer’s comment and fully acknowledge that paradigm shift and precision targeting are overstated due to the lack of experimental validation. These terms have been completely removed from the revised manuscript, and all relevant descriptions have been replaced with cautious, objective wording that matches the exploratory bioinformatic nature of our study, with no overinterpretation of the findings.

19. Drug names, gene names, and abbreviations are inconsistently written (e.g., PHENCYYCLIDINE, spacing errors, punctuation issues).

We thank the reviewer for this careful comment. We have carefully checked and unified the writing format of all drug names, gene names, and abbreviations throughout the manuscript, including correcting spelling errors (e.g., phencyclidine), inconsistent capitalization, spacing mistakes, and punctuation issues. All terms are now presented in a consistent and standardized manner.

20. Potential risks, translational barriers, and clinical feasibility of using exosomal gene models are not discussed.

We sincerely appreciate the reviewer’s valuable comment. We acknowledge that the potential risks, translational barriers, and clinical feasibility of using the exosome gene model for AD prediction were not sufficiently discussed in the original manuscript. These critical points have been comprehensively added and elaborated in the revised Discussion section, including safety considerations, technical challenges in clinical application, ethical constraints, and practical obstacles in clinical translation. We have also provided objective and balanced interpretations of the clinical applicability of the current model, fully considering its advantages and limitations.

To reviewer 2

Reviewer #2: Overall, the study addresses an interesting and relevant topic. With clearer methodological descriptions and more cautious interpretation of the findings, the manuscript could be significantly strengthened.

Major Comments

• The manuscript refers to “exosomal genes,” but the datasets used are bulk transcriptomic datasets rather than samples derived from isolated exosomes. It would help if the authors clarified how these genes were defined as exosome-related and provided stronger justification for linking them to exosomal biology in Alzheimer’s disease.

Thank you for your valuable comment. We clarify that the bulk transcriptomic datasets used in this study were from the GEO database, with all samples being human brain tissue of AD patients and normal controls. We defined exosome-related genes via dual validation: intersecting AD differential genes from the above brain tissue bulk data with exosome-related genes (relevance score ≥ 2) from the GeneCards database. Moreover, published studies have confirmed that our identified 5 core genes are enriched in exosomes secreted by brain CNS cells and participate in AD exosomal biological processes. Relevant screening details and literature citations have been supplemented in the revised manuscript (marked in red). We thank you again for your comment that improved the rigor of our study.

• Some important methodological details are missing. In particular, the manuscript should clearly describe how batch effects between the GEO da

Attachment Submitted filename: Response to Reviewers.docx

# Peer review: Decision Letter 1

**DOI:** 10.1371/journal.pone.0354014.r003

**Authors:** Amr Ahmed El-Arabey, Amr Ahmed El-Arabey

29 Apr 2026

PONE-D-25-47921R1

Exosomal Gene-Based Predictive Model and Therapeutic Target Identification for Alzheimer's Disease: A Bioinformatics Analysis

PLOS One

Dear Dr. Ma,

Thank you for submitting your manuscript to PLOS ONE. After careful consideration, we feel that it has merit but does not fully meet PLOS ONE’s publication criteria as it currently stands. Therefore, we invite you to submit a revised version of the manuscript that addresses the points raised during the review process.

Please submit your revised manuscript by Jun 12 2026 11:59PM. If you will need more time than this to complete your revisions, please reply to this message or contact the journal office at plosone@plos.org. When you're ready to submit your revision, log on to https://www.editorialmanager.com/pone/ and select the 'Submissions Needing Revision' folder to locate your manuscript file.

Please include the following items when submitting your revised manuscript:

- A letter that responds to each point raised by the academic editor and reviewer(s). You should upload this letter as a separate file labeled 'Response to Reviewers'.
- A marked-up copy of your manuscript that highlights changes made to the original version. You should upload this as a separate file labeled 'Revised Manuscript with Track Changes'.
- An unmarked version of your revised paper without tracked changes. You should upload this as a separate file labeled 'Manuscript'.

If you would like to make changes to your financial disclosure, please include your updated statement in your cover letter. Guidelines for resubmitting your figure files are available below the reviewer comments at the end of this letter.

If applicable, we recommend that you deposit your laboratory protocols in protocols.io to enhance the reproducibility of your results. Protocols.io assigns your protocol its own identifier (DOI) so that it can be cited independently in the future. For instructions see: https://journals.plos.org/plosone/s/submission-guidelines#loc-laboratory-protocols. Additionally, PLOS ONE offers an option for publishing peer-reviewed Lab Protocol articles, which describe protocols hosted on protocols.io. Read more information on sharing protocols at https://plos.org/protocols?utm_medium=editorial-email&utm_source=authorletters&utm_campaign=protocols.

As the corresponding author, your ORCID iD is verified in the submission system and will appear in the published article. PLOS supports the use of ORCID, and we encourage all coauthors to register for an ORCID iD and use it as well. Please encourage your coauthors to verify their ORCID iD within the submission system before final acceptance, as unverified ORCID iDs will not appear in the published article. *Only*  the individual author can complete the verification step; PLOS staff *cannot*  verify ORCID iDs on behalf of authors.

We look forward to receiving your revised manuscript.

Kind regards,

Amr Ahmed El-Arabey

Academic Editor

PLOS One

Journal Requirements:

If the reviewer comments include a recommendation to cite specific previously published works, please review and evaluate these publications to determine whether they are relevant and should be cited. There is no requirement to cite these works unless the editor has indicated otherwise.

Please review your reference list to ensure that it is complete and correct. If you have cited papers that have been retracted, please include the rationale for doing so in the manuscript text, or remove these references and replace them with relevant current references. Any changes to the reference list should be mentioned in the rebuttal letter that accompanies your revised manuscript. If you need to cite a retracted article, indicate the article’s retracted status in the References list and also include a citation and full reference for the retraction notice.

[Note: HTML markup is below. Please do not edit.]

Reviewers' comments:

Reviewer's Responses to Questions

**Comments to the Author**

1. If the authors have adequately addressed your comments raised in a previous round of review and you feel that this manuscript is now acceptable for publication, you may indicate that here to bypass the “Comments to the Author” section, enter your conflict of interest statement in the “Confidential to Editor” section, and submit your "Accept" recommendation.

Reviewer #1: All comments have been addressed

Reviewer #2: All comments have been addressed

**********

2. Is the manuscript technically sound, and do the data support the conclusions?

The manuscript must describe a technically sound piece of scientific research with data that supports the conclusions. Experiments must have been conducted rigorously, with appropriate controls, replication, and sample sizes. The conclusions must be drawn appropriately based on the data presented.

Reviewer #1: Yes

Reviewer #2: Yes

**********

3. Has the statistical analysis been performed appropriately and rigorously?

Reviewer #1: Yes

Reviewer #2: Yes

**********

4. Have the authors made all data underlying the findings in their manuscript fully available?

The PLOS Data policy requires authors to make all data underlying the findings described in their manuscript fully available without restriction, with rare exception (please refer to the Data Availability Statement in the manuscript PDF file). The data should be provided as part of the manuscript or its supporting information, or deposited to a public repository. For example, in addition to summary statistics, the data points behind means, medians and variance measures should be available. If there are restrictions on publicly sharing data—e.g. participant privacy or use of data from a third party—those must be specified.

Reviewer #1: Yes

Reviewer #2: Yes

**********

5. Is the manuscript presented in an intelligible fashion and written in standard English?

PLOS ONE does not copyedit accepted manuscripts, so the language in submitted articles must be clear, correct, and unambiguous. Any typographical or grammatical errors should be corrected at revision, so please note any specific errors here.

Reviewer #1: Yes

Reviewer #2: Yes

**********

6. Review Comments to the Author

Please use the space provided to explain your answers to the questions above. You may also include additional comments for the author, including concerns about dual publication, research ethics, or publication ethics. (Please upload your review as an attachment if it exceeds 20,000 characters)

Reviewer #1: The authors have clearly tried to respond to the reviewers, but many of their answers feel a bit generic and don’t explain exactly what was changed. While they did improve some important points like toning down strong claims and fixing a few scientific inaccuracies; some key issues, especially around methods and validation, are still not clearly or consistently addressed. Overall, it’s a step in the right direction, but the responses could be more specific and transparent to fully convince the reviewers.

Reviewer #2: The manuscript is well-conducted and has improved significantly after revision. The integration of multiple datasets with machine learning approaches is appropriate and adds strength to the analysis . The methodology is now clearly described, including normalization, batch correction, and validation, which improves reproducibility. The authors have also moderated their claims, presenting the model realistically with moderate predictive performance, and have expanded the biological interpretation while acknowledging key limitations such as the lack of external validation. Only minor issues remain, mainly related to slight language polishing, small formatting inconsistencies, and briefly clarifying docking details in the main text. Overall, the study is scientifically sound and suitable for publication.

**********

7. PLOS authors have the option to publish the peer review history of their article (what does this mean?). If published, this will include your full peer review and any attached files.

If you choose “no”, your identity will remain anonymous but your review may still be made public.

**Do you want your identity to be public for this peer review?**  For information about this choice, including consent withdrawal, please see our Privacy Policy.

Reviewer #1: No

Reviewer #2: **Yes:** DIBYASHREE CHHETRI

**********

[NOTE: If reviewer comments were submitted as an attachment file, they will be attached to this email and accessible via the submission site. Please log into your account, locate the manuscript record, and check for the action link "View Attachments". If this link does not appear, there are no attachment files.]

To ensure your figures meet our technical requirements, please review our figure guidelines: https://journals.plos.org/plosone/s/figures

You may also use PLOS’s free figure tool, NAAS, to help you prepare publication quality figures: https://journals.plos.org/plosone/s/figures#loc-tools-for-figure-preparation.

NAAS will assess whether your figures meet our technical requirements by comparing each figure against our figure specifications.

# Author comment: Author response to Decision Letter 2

**DOI:** 10.1371/journal.pone.0354014.r004

30 Apr 2026

Dear Editor and Reviewers,

We greatly appreciate the editors and reviewers for their valuable and constructive comments, which have significantly improved the quality of our manuscript. We have carefully addressed all concerns, and detailed point-by-point responses are provided below.

Response to Reviewer 1

We sincerely appreciate your rigorous and thoughtful review. We fully acknowledge that our previous responses were relatively general and lacked specific descriptions of the exact revisions made in the manuscript. To fully address your concerns regarding methodological transparency, reproducibility, validation rigor, and consistency, we have now completely and thoroughly revised the manuscript, with all changes clearly marked in the tracked version. We have supplemented every key step with explicit, step-by-step details to ensure full clarity and scientific rigor. Our point-by-point response is as follows:

1. The title contained too many concepts and was difficult to follow.

Response:

We have simplified and refined the title to highlight the core focus.

Revised title:

Exosomal Gene-Based Predictive Model and Therapeutic Target Identification for Alzheimer's Disease: A Bioinformatics Analysis

2. Repetitive descriptions of exosomes across sections.

Response:

We have streamlined redundant descriptions and retained only essential background information. Mechanistic insights and translational implications are concentrated in the Discussion to improve logical flow and readability.

3. Grammatical errors, awkward sentences, and inconsistent phrasing.

Response:

The entire manuscript has been professionally edited to correct grammatical errors, optimize sentence structure, and ensure consistent terminology throughout.

4. Key methodological steps missing (normalization, batch correction, validation); study not reproducible.

Response:

We have added complete, explicit, step-by-step protocols in the Methods section, including:

Data normalization (log₂ transformation + between-array normalization)

Batch effect correction using the ComBat algorithm

Full model validation (10-fold cross-validation, calibration, DCA)

All analysis code is provided as supplementary files to ensure full reproducibility.

5. Four GEO datasets from different sources; no clear batch effect handling.

Response:

We have added clear descriptions: batch effects across datasets were eliminated using the ComBat function from the sva package. PCA plots before and after correction are presented to confirm successful removal of batch bias. Corresponding code is available in SVA.txt.

6. Unclear definition/filtering of exosomal genes from GeneCards; no cutoff stated.

Response:

Exosome-related genes were retrieved from GeneCards using the keyword “exosomes” with a strict cutoff of relevance score ≥ 2. This criterion is clearly stated in the Methods.

7. Choice of LASSO, SVM, random forest not justified; parameters not reported.

Response:

We have added detailed justification and complete parameters:

LASSO: feature dimension reduction; 10-fold cross-validation; λ selected by minimum error

SVM-RFE: identify optimal gene sets with minimum cross-validation error

Random Forest: ntree = 500; gene importance scored by MeanDecreaseGini

All justifications and code files are clearly reported.

8. Same dataset used for feature selection and model evaluation, which may inflate performance.

Response:

We have adopted 10-fold cross-validation and independent train-test splitting to completely separate feature selection and model evaluation, eliminating data leakage and overfitting. All results were updated accordingly.

9. AUC > 0.7 described as “robust”; overstated.

Response:

We have removed “robust” and revised to moderate and stable predictive performance (AUC > 0.70), consistent with the exploratory bioinformatic nature of this study.

10. No external validation; limited clinical reliability.

Response:

We fully acknowledge this limitation. Owing to the scarcity of well-annotated AD brain tissue datasets under ethical constraints, we performed rigorous internal validation including bootstrap resampling, 10-fold cross-validation, calibration, and DCA. Multi-center external validation will be conducted in future studies.

11. Insufficient mechanistic analysis of CD44, CXCR4, TUBB, PSMA5, PSMB3 in AD.

Response:

We have added a comprehensive mechanistic discussion for each gene in AD pathogenesis, including their roles in neuroinflammation, Aβ clearance, tau pathology, cytoskeleton stability, proteasome function, and circadian regulation, supported by relevant published literature.

12. Some identified compounds (phencyclidine, thioridazine) are neurotoxic or clinically inappropriate; not discussed.

Response:

We have added critical safety and clinical suitability discussions. These compounds are included only as molecular probes, not for direct clinical use, and their potential risks are clearly addressed.

13. Docking targets listed only as residues without protein structure or binding context.

Response:

We have supplemented corresponding PDB IDs, functional domains, and binding environments for all docking residues. Details are included in the manuscript and supplementary materials.

14. The study is entirely computational; no experimental validation.

Response:

We recognize this as a major limitation and clearly state it in the manuscript. This study provides exploratory computational predictions; experimental validation will be performed in future work.

15. Figures cited but key messages not clearly described in the text.

Response:

We have added clear biological interpretations for all figures (1–9) in the Results section. Each panel is explained with its core finding and implication.

16. Immune cell correlations reported but biological meaning in AD not explored.

Response:

We have added detailed discussion linking the 5-gene signature to immune cells (B cells, CD4+/CD8+ T cells, MDSCs) and AD neuroinflammation, amyloid deposition, and tau hyperphosphorylation.

17. Discussion mainly restates results rather than providing critical interpretation.

Response:

We have completely rewritten the Discussion to emphasize mechanistic insights, translational value, literature comparison, and in-depth limitations, rather than only summarizing results.

18. Overstated claims such as “paradigm shift” and “precision targeting.”

Response:

All exaggerated statements have been removed. Conclusions are now cautious, objective, and consistent with a computational bioinformatics study.

19. Inconsistent formatting of drug/gene/abbreviation names (e.g., PHENCYYCLIDINE).

Response:

All gene symbols, drug names, abbreviations, and spellings have been carefully checked and standardized. “Phencyclidine” is now correctly used throughout.

20. Potential risks, translational barriers, and clinical feasibility not discussed.

Response:

We have added a dedicated section discussing clinical translation challenges: exosome isolation standardization, cost, ethical constraints, non-specific signals, and implementation hurdles.

We greatly appreciate your valuable and professional guidance, which has significantly improved the quality and rigor of our manuscript. We sincerely hope these detailed, transparent, and comprehensive revisions fully address all remaining concerns.

Response to Reviewer 2

We greatly appreciate your positive and constructive feedback. We are delighted that our revisions have met your approval. As suggested, we have:

Further polished the English language and fluency of the entire manuscript.

Corrected all minor formatting inconsistencies throughout the text.

Briefly clarified the details of molecular docking (including binding residues and structural context) in the main text.

Thank you again for your valuable comments, which have greatly improved the presentation of our work.

Attachment Submitted filename: Response_to_Reviewers_auresp_2.docx

# Peer review: Decision Letter 2

**DOI:** 10.1371/journal.pone.0354014.r005

**Authors:** Amr Ahmed El-Arabey, Amr Ahmed El-Arabey, Amr Ahmed El-Arabey

2 Jul 2026

Exosomal Gene-Based Predictive Model and Therapeutic Target Identification for Alzheimer's Disease: A Bioinformatics Analysis

PONE-D-25-47921R2

Dear Dr. Ma,

We’re pleased to inform you that your manuscript has been judged scientifically suitable for publication and will be formally accepted for publication once it meets all outstanding technical requirements.

Within one week, you’ll receive an e-mail detailing the required amendments. When these have been addressed, you’ll receive a formal acceptance letter and your manuscript will be scheduled for publication.

An invoice will be generated when your article is formally accepted. Please note, if your institution has a publishing partnership with PLOS and your article meets the relevant criteria, all or part of your publication costs will be covered. Please make sure your user information is up-to-date by logging into Editorial Manager at Editorial Manager® and clicking the ‘Update My Information' link at the top of the page. For questions related to billing, please contact billing support.

If your institution or institutions have a press office, please notify them about your upcoming paper to help maximize its impact. If they’ll be preparing press materials, please inform our press team as soon as possible -- no later than 48 hours after receiving the formal acceptance. Your manuscript will remain under strict press embargo until 2 pm Eastern Time on the date of publication. For more information, please contact onepress@plos.org.

Kind regards,

Amr Ahmed El-Arabey

Academic Editor

PLOS One

Additional Editor Comments (optional):

Reviewers' comments:

Reviewer's Responses to Questions

**Comments to the Author**

1. If the authors have adequately addressed your comments raised in a previous round of review and you feel that this manuscript is now acceptable for publication, you may indicate that here to bypass the “Comments to the Author” section, enter your conflict of interest statement in the “Confidential to Editor” section, and submit your "Accept" recommendation.

Reviewer #1: All comments have been addressed

**********

2. Is the manuscript technically sound, and do the data support the conclusions?

The manuscript must describe a technically sound piece of scientific research with data that supports the conclusions. Experiments must have been conducted rigorously, with appropriate controls, replication, and sample sizes. The conclusions must be drawn appropriately based on the data presented.

Reviewer #1: Yes

**********

3. Has the statistical analysis been performed appropriately and rigorously?

Reviewer #1: Yes

**********

4. Have the authors made all data underlying the findings in their manuscript fully available?

The PLOS Data policy requires authors to make all data underlying the findings described in their manuscript fully available without restriction, with rare exception (please refer to the Data Availability Statement in the manuscript PDF file). The data should be provided as part of the manuscript or its supporting information, or deposited to a public repository. For example, in addition to summary statistics, the data points behind means, medians and variance measures should be available. If there are restrictions on publicly sharing data—e.g. participant privacy or use of data from a third party—those must be specified.

Reviewer #1: Yes

**********

5. Is the manuscript presented in an intelligible fashion and written in standard English?

PLOS ONE does not copyedit accepted manuscripts, so the language in submitted articles must be clear, correct, and unambiguous. Any typographical or grammatical errors should be corrected at revision, so please note any specific errors here.

Reviewer #1: Yes

**********

6. Review Comments to the Author

Please use the space provided to explain your answers to the questions above. You may also include additional comments for the author, including concerns about dual publication, research ethics, or publication ethics. (Please upload your review as an attachment if it exceeds 20,000 characters)

Reviewer #1: The manuscript is technically sound, the methodology is adequately described, and the data support the conclusions drawn by the authors. The statistical analyses appear appropriate, and the results are presented clearly. The manuscript is well written and understandable, and the data availability requirements have been satisfactorily addressed.

**********

7. PLOS authors have the option to publish the peer review history of their article (what does this mean?). If published, this will include your full peer review and any attached files.

If you choose “no”, your identity will remain anonymous but your review may still be made public.

**Do you want your identity to be public for this peer review?**  For information about this choice, including consent withdrawal, please see our Privacy Policy.

Reviewer #1: No

**********

# Editor report: Acceptance letter

**DOI:** 10.1371/journal.pone.0354014.r006

**Authors:** Amr Ahmed El-Arabey, Amr Ahmed El-Arabey, Amr Ahmed El-Arabey

PONE-D-25-47921R2

PLOS One

Dear Dr. Ma,

I'm pleased to inform you that your manuscript has been deemed suitable for publication in PLOS One. Congratulations! Your manuscript is now being handed over to our production team.

At this stage, our production department will prepare your paper for publication. This includes ensuring the following:

* All references, tables, and figures are properly cited

* All relevant supporting information is included in the manuscript submission,

* There are no issues that prevent the paper from being properly typeset

You will receive further instructions from the production team, including instructions on how to review your proof when it is ready. Please keep in mind that we are working through a large volume of accepted articles, so please give us a few days to review your paper and let you know the next and final steps.

Lastly, if your institution or institutions have a press office, please let them know about your upcoming paper now to help maximize its impact. If they'll be preparing press materials, please inform our press team within the next 48 hours. Your manuscript will remain under strict press embargo until 2 pm Eastern Time on the date of publication. For more information, please contact onepress@plos.org.

You will receive an invoice from PLOS for your publication fee after your manuscript has reached the completed accept phase. If you receive an email requesting payment before acceptance or for any other service, this may be a phishing scheme. Learn how to identify phishing emails and protect your accounts at https://explore.plos.org/phishing.

If we can help with anything else, please email us at customercare@plos.org.

Thank you for submitting your work to PLOS ONE and supporting open access.

Kind regards,

PLOS ONE Editorial Office Staff

on behalf of

Dr. Amr Ahmed El-Arabey

Academic Editor

PLOS One
